# Quadruplex DNA in long terminal repeats in maize LTR retrotransposons inhibits the expression of a reporter gene in yeast

**DOI:** 10.1186/s12864-018-4563-7

**Published:** 2018-03-06

**Authors:** Viktor Tokan, Janka Puterova, Matej Lexa, Eduard Kejnovsky

**Affiliations:** 1 Department of Plant Developmental Genetics, Institute of Biophysics, Czech Academy of Sciences, Kralovopolska 135, 61200 Brno, Czech Republic; 2 Department of Information Systems, Faculty of Information Technology, Brno University of Technology, 61200 Brno, Czech Republic; 3 Faculty of Informatics, Masaryk University, Botanicka 68a, 60200 Brno, Czech Republic

**Keywords:** G4 motifs, Quadruplex DNA, Transposable elements, Maize LTR retrotransposons, Circular dichroism, NMM ligand

## Abstract

**Background:**

Many studies have shown that guanine-rich DNA sequences form quadruplex structures (G4) in vitro but there is scarce evidence of guanine quadruplexes in vivo. The majority of potential quadruplex-forming sequences (PQS) are located in transposable elements (TEs), especially close to promoters within long terminal repeats of plant LTR retrotransposons.

**Results:**

In order to test the potential effect of G4s on retrotransposon expression, we cloned the long terminal repeats of selected maize LTR retrotransposons upstream of the lacZ reporter gene and measured its transcription and translation in yeast. We found that G4s had an inhibitory effect on translation in vivo since “mutants” (where guanines were replaced by adenines in PQS) showed higher expression levels than wild-types. In parallel, we confirmed by circular dichroism measurements that the selected sequences can indeed adopt G4 conformation in vitro. Analysis of RNA-Seq of polyA RNA in maize seedlings grown in the presence of a G4-stabilizing ligand (NMM) showed both inhibitory as well as stimulatory effects on the transcription of LTR retrotransposons.

**Conclusions:**

Our results demonstrate that quadruplex DNA located within long terminal repeats of LTR retrotransposons can be formed in vivo and that it plays a regulatory role in the LTR retrotransposon life-cycle, thus also affecting genome dynamics.

**Electronic supplementary material:**

The online version of this article (10.1186/s12864-018-4563-7) contains supplementary material, which is available to authorized users.

## Background

Guanine-rich sequence motifs with four closely spaced runs of Gs are able to form a four-stranded structure known as a G-quadruplex (G4, for review see [1]). Quadruplexes can be formed by both DNA and RNA molecules, are stabilized by potassium or sodium ions and can adopt various conformations involving one, two or four molecules [2]. Recent genome-wide in silico studies revealed that genomes contain thousands of G4 motifs which are enriched in certain loci, as seen in the human [3, 4] and maize [5]. The highest occurrences of G4 motifs have been observed at the telomeres, origins of replication, promoters, translational start sites, 5′ and 3′ UTRs, and intron-exon boundaries, thus suggesting specific molecular/biological functions. A regulatory roles of DNA and RNA G-quadruplexes were summarized recently by several comprehensive reviews [6, 7].

Many studies have shown that guanine-rich sequences form quadruplex DNA or RNA in vitro but solid experimental evidence of quadruplex formation in vivo has been gathered only recently (for review see [6, 7]) although many quadruplexes that are formed in vitro are unfolded in living cells [8]. This research was greatly aided by the development and use of small chemical ligands to stabilize the G4s [9] as well as a single chain antibody specific for G4s [10].

While in general the biggest focus is on genic and telomeric G4 motifs, the majority of G4 motifs are however localized in the repetitive fraction of genomes. For example, in the maize genome, mostly composed of LTR retrotransposons, 71% of non-telomeric G4 motifs are located in repetitive genomic regions [5]. Lexa et al. [11] analysed 18,377 LTR retrotransposons from 21 plant species and found that PQS are frequently present within LTRs, more often at specific distances from other regulatory elements such as transcription start sites. Moreover, evolutionarily younger and active elements of plants and human had more PQS, altogether indicating that G4s can play a role in the LTR retrotransposon life cycle [11, 12]. In addition, recent study has shown that quadruplexes localized within the 3’UTR of LINE-1 elements can stimulate retrotransposition [13].

Currently a range of tools exist for detection of potential quadruplex forming sites in genomes. While most look for clusters of G runs in DNA sequences with constrained spacing and use regular expressions or recursive searches (e.g. quadparser [3], QGRS MApper [14], pqsfinder [15]) other evaluate G-richness and G-skewness in a sliding window (G4Hunter [16]) or use machine learning based on broadly defined sequence composition [17, 18]. While the former have the advantage of having intuitive parameters and describing better the topology and intramolecular binding in the potential quadruplex, the latter have more parameters and may possibly be tuned to higher sensitivity, although it isn’t clear this is currently the case, as seen in comparisons in [15].

Here we show that the presence of G4 motifs within maize LTRs results in a markedly reduced expression of the downstream located lacZ gene in yeast compared to a similar sequence with mutations preventing quadruplex formation. Additionally, our results suggest that G4 formation affects translation rather than transcription, in a strand-specific manner.

## Methods

### TE reference sequence annotation

All LTR retroelement sequences were downloaded from Maize Transposable Element Database (http://maizetedb.org/~maize/) and searched for G4 motifs using the R/Bioconductor [19] package *pqsfinder* [15]. Pqsfinder searches for clusters of guanines in nucleic acid sequences that satisfy a set of biologically and chemically relevant constraints. These include number of guanines in a single guanine run (minimum 2), distance between the runs (or loop length) and its variability within the quadruplex as well as the number of mismatches and bulges present in the potential quadruplex sequence which tend to destabilize the structure. The cited work found parametrization of these criteria that corresponded best to G4-seq sequencing data by Chambers et al. [20]. In a very crude approximation, a single mismatch, bulge or an extremely long loop will counter the stabilization effect of an extra guanine tetrad. Default settings were used, except for the minimum score value. A value of 65 was used when fewer false positive results were desirable. LTRs were predicted by LTR finder [21]. BLASTX [22] was used against a collection of TE protein sequences downloaded from GypsyDB [23] with e-value threshold set to 0.01 to generate annotations in the Additional file 1. For LTR amplification ZMMBBc library (also reffered as CHORI201) BAC clones containing selected elements were ordered from the Arizona Genomics Institute. Additional table shows selected elements and coresponding BAC clones used for the yeast in vivo assay (see Additional file 2).

### CD measurements and polyacrylamide gel electrophoresis

Circular dichroism and polyacrylamide gel electrophoresis were performed as described in Lexa et al. [11] but with the temperature at 27 °C in accordance with to yeast growth conditions. Sequences of oligonucleotides used for CD measurments are listed in Additional file 2.

### Cloning and mutagenesis

We used the pESC-URA plasmid (Agilent) as the backbone for our constructs. The Gal1 promoter was excised through SpeI/XhoI digestion and a p424 SpeI/XhoI fragment containing MCS was cloned in [24]. We used the following primers and Q5 polymerase (NEB) for lacZ coding sequence amplification from *E. coli* (K12) genomic DNA:

lacZ_F ATCGTCGACATGACCATGATTACGGATTCACTGG and lacZ_R CCTGTCGACTTATTTTTGACACCAGACCAACTGG. Both primers have SalI extension which was used for lacZ cloning, with the orientation being verified by PCR and sequencing. A list of primers used for LTRs amplification is in Additional file 2. LTRs were amplified using Q5 polymerase under the recommended conditions and blunt cloned into the SmaI site of pBC. Again the insertions were verified by PCR and sequencing. Mutations in G4 forming sequences in cloned LTRs were introduced using single mutagenic primers for each LTR and Q5 polymerase (recommended conditions, Additional file 2). The products were treated with DpnI (NEB) and 1 μl was used for XL-1 blue electrocompetent cell (Agilent) transformation. Mutations were verified by sequencing.

### Yeast lacZ assay

We used the *S. cerevisiae* strain CM100 (MATα, can1–100 oc, his3, leu2, trp1, ura3–52) for the lacZ expression assay. Vectors containing lacZ under control of LTR promoter were transformed into yeast using S.C. Easy Comp Transformation Kit (Invitrogen). Transformed cells were plated on selective media without Uracil. For each construct we measured lacZ expression as follows. Six colonies were inoculated into 500 μl liquid media in a deep-well plate and grown overnight (cca 20 h) at 28 °C / 250 rpm. The next day 150 μl culture was transferred into 1500 μl new media and cultivated overnight at 28 °C / 250 rpm. The following morning the OD_600_ of the culture was about 1. We transferred 200 μl of the culture into a 96-well microplate and centrifuged to collect the cells, discarded 190 μl of the supernatant, resuspended the cells and permeabilized them for 15 min at 30 °C / 250 rpm in 110 μl modified Z-buffer (100 mM Na_2_HPO_4_, 40 mM Na_2_H_2_PO_4_, 10 mM KCl, 2 mM MgSO_4_, 0.1% SDS). Next 25 μl of 4,17 nM ONPG was added and the plate incubated at 30 °C/ 250 rpm. When a pale yellow colour developed the reaction was ceased using 135 μl stop solution (1 M Na_2_CO_3_). The plate was centrifuged and clear supernatant was used for reading Abs_420_ (both Abs_420_ and OD_600_ were measured using a Tecan Sunrise microplate reader with Rainbow filter). For the starting value of Abs_420_ we used a well where no cells were added and so autolysis of ONPG was included. LacZ units were calculated using the formula: lacZ units = 1000 * (Abs_420_ / (OD_600_ * volume [ml] * time [min]). Each plasmid was tested in triplicate. We averaged measurements for each colony and used ANOVA (*p* > 0.001) and post-hoc Tukey HSD to compare lacZ units in different construct pairs (wt vs mutant).

### Yeast RNA isolation and Q-PCR

Yeast for RNA isolation were grown the same way as for lacZ assay but for the final day the whole volume was used. RNA was prepared by extraction with hot acidic phenol [25] and then treated with TURBO DNase (Ambion). Reverse transcription was carried out using a High-Capacity RNA-to-cDNA kit (Applied Biosystems) and Q-PCR was performed using a SensiFAST SYBR Hi-ROX kit (Bioline). We used 2 pairs of primers, first for lacZ as gene of interest (qlacZ_F GAAAGCTGGCTACAGGAAG; qlacZ_R GCAGCAACGAGACGTCA) and second for URA marker as reference gene (qURA3_F GGATGTTCGTACCACCAAGG; qURA3_R TGTCTGCCCATTCTGCTATT).

### Transcription start sites prediction and rapid cDNA ends amplification (RACE)

Transcriptional start sites (TSS) were predicted using TSSPlant [26]. Experimental verification of TSS was performed with SMARTer™ RACE cDNA Amplification Kit (Clontech) using total RNA from yeast and maize (B73) respectively, which were isolated as described herein. Primers used for RACE are listed in Additional file 2. Products were cloned into pCR™II Vector (Invitrogen) and transformed into One Shot™ TOP10 *E. coli* electrocompetent cells (Invitrogen), 8 colonies were sequenced.

### Plant material preparation

*Zea mays* B73 seeds were obtained from the U.S. National Plant Germplasm System (https://npgsweb.ars-grin.gov). Seeds were sterilized and germinated in moisturized filter papers for 5 days at room temperature. 5th day seedlings were transferred to ¼ concentration of aerated Reid-York solution [27] in a greenhouse. Each seedling was secured by plastic foam strip in separate 50 ml falcon tube and positions of NMM treated and non-treated plants were randomized, solution was changed on daily basis. After 2 and 4 days the solution was replaced by ½ and full concentration, respectively. Treatment by 16 μM NMM (Frontier Scientific) commenced after 1 day growth in full Reid-York solution concentration and continued for 3 days. After 3 days of NMM treatment, the roots of 4 treated and 4 non-treated plants were used for RNA isolation by NucleoSpin® RNA Plant kit (Machery-Nagel).

### cDNA library preparation and RNA sequencing

In total eight RNA samples (2 μg each) were provided to the Genomics Core Facility Center (EMBL Heidelberg) for the construction of cDNA libraries with poly(A) + selection and sequencing. Sequencing libraries were prepared using an ILMN truseq stranded mRNA Kit (Illumina, San Diego, CA, USA) according to manufacturer’s protocol. Sequencing libraries were pooled in equimolar concentration and sequenced on an Illumina NextSeq 500, producing 2 × 80-nucleotide paired-end reads.

### RNA-Seq quality control and preprocessing

Raw RNA-Seq libraries contained 47–56 million paired-end reads for treated samples and 47–62 million paired-end reads for control samples. Reads were checked for quality using FastQC ([28], available online at: http://www.bioinformatics.babraham.ac.uk/projects/fastqc). Reads with low-quality, containing adaptor sequences, unpaired reads, containing rRNA contamination (18S rRNA - GenBank: AF168884.1, 26S rRNA - GenBank: NR_028022.2, 5.8S rRNA - GenBank: U46603.1) and reads containing poly-G runs, which are a typical error for NextSeq platform, were removed using Trimmomatic 0.36 [29] and trimmed to 75 bp length. After preprocessing, read libraries ranged between 17 and 35 million paired-end reads for treated samples and 14–45 million paired-end reads for control samples. In order to obtain more consistent results, the smallest libraries were discarded from both groups, giving libraries ranging from 30 to 35 million paired-end reads for treated samples and from 33 to 45 million paired-end reads control samples. RNA-Seq data was deposited in the European Nucleotide Archive ENA under primary accession number: PRJEB23390. To find out if there was any contamination in the reads, they were mapped onto the maize reference genome B73 RefGen_v3 (ftp://ftp.ensemblgenomes.org/pub/plants/release-31/fasta/zea_mays/dna/) using STAR aligner v2.5.2b [30] with default settings. For all libraries, more than 95% of reads mapped onto the reference genome, indicating that there was no significant contamination.

### Mapping RNA-Seq on library of transposable elements and their differential expression analysis

To estimate the expression of individual maize transposable elements, RNA-Seq reads were mapped using STAR aligner v2.5.2b [30] on the Maize transposable elements database (http://maizetedb.org). Due to a differences in mapping reads onto transposable elements (multiple copies in genome, sequence variability of transposons falling into same family/subfamily, less variable length) compared to onto genes, we adjusted mapping settings to allow multimaps and a higher number of mismatches in mapped reads to reflect transposon variability: *--winAnchorMultimapNmax* 1000, *−-outFilterMultimapNmax* 1000, *−-outFilterMismatchNmax* 15, *−-alignIntronMin* 5 *--alignIntronMax* 20,000. The number of mapped reads with these settings varied from 234 to 360 thousand, corresponding to 0.68–1.05% of library sizes. Subsequently, to obtain raw counts of mapped reads per transposable element, the *featureCounts* [31] tool with *--fraction* option was used to assign counts of multi-mapped reads to transposons correctly and to avoid multiple counts of the same sequence. These raw counts were used for differential expression analysis performed with the *EdgeR* package [32], which is recommended to use for smaller numbers of biological replicates [33]. Poorly expressed transposons which had count-per-million (CPM) figures of less than 45 in at least three samples (corresponding to 10–12 reads mapped onto transposons) were removed from further analysis. The statistical values (log fold change (LFC), *p*-value) were estimated using the *exactTest* function and adjusted *p*-values (FDR) with the *p.adjust* function. Transposons with LFC > | 1.5 | and FDR < 0.05 were considered as differentially expressed. Such transposons were annotated as described above in the TE reference sequence annotation section. Elements with inconsistecies in annotation, e.g. wrong order of protein domains, were excluded from the analysis. To correlate RNA-Seq coverage with position of quadruplexes in differentially expressed LTR retrotransposons, RNA-Seq coverage was estimated by *bedtools genomecov* [34] with settings *-d -split -scale $norm_factor*, where *$norm_factor* represents normalization factor calculated for each library by the *EdgeR* package. RNA-Seq coverage for all control and treatment samples was aggregated to average coverage and plotted by using custom R script together with annotation of LTR retrotransposons.

## Results

### Selection of maize LTR retrotransposons with PQS and confirmation of quadruplex formation by circular dichroism

**Fig. 1:**
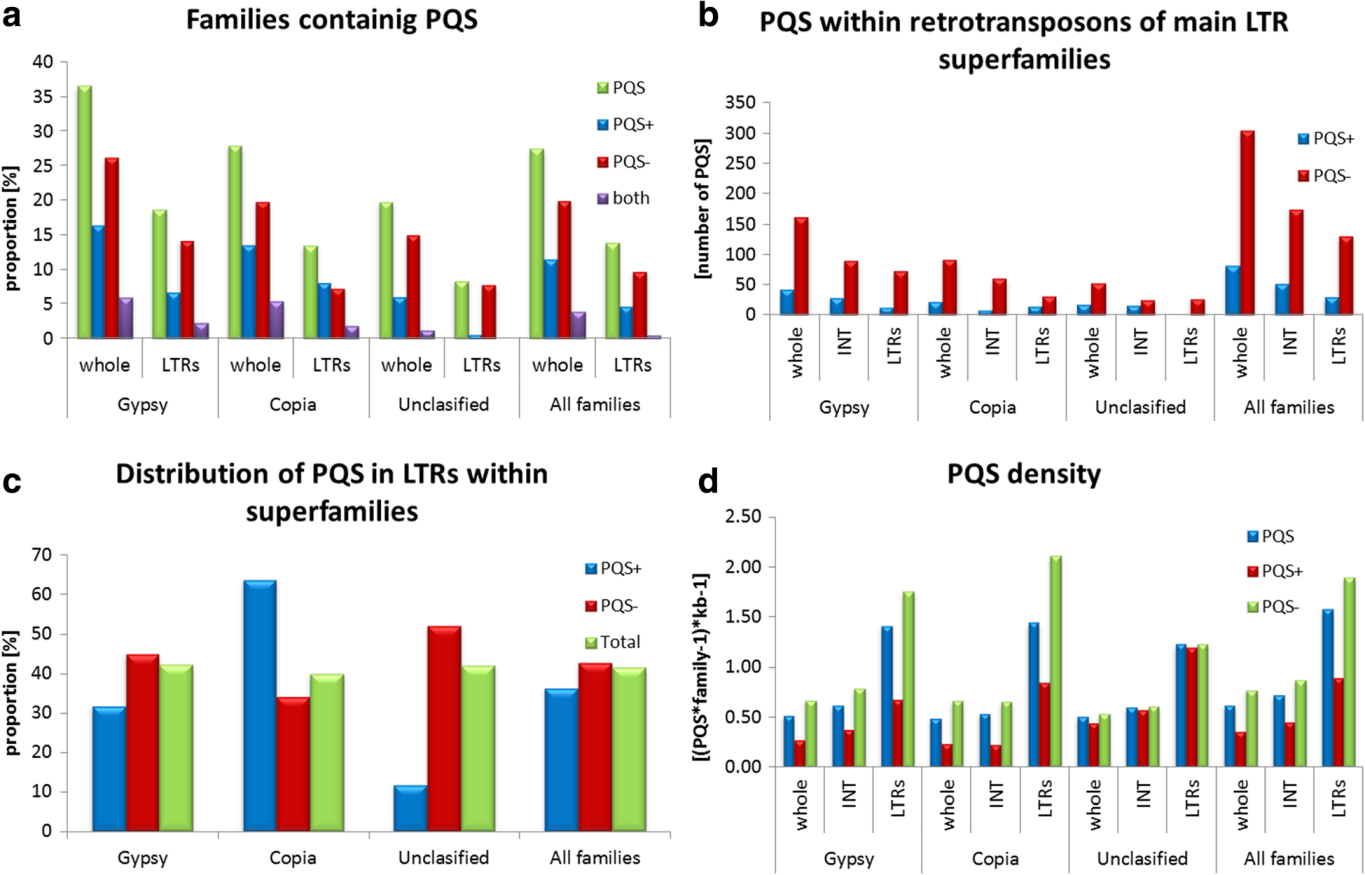
Comparison of different retrotransposon superfamilies and in silico predicted potential quadruplex-forming sequences (PQS). **a** Chart shows proportion of families that possess at least one PQS (green) PQS located on plus (PQS+; blue) minus (PQS-; red) and both strands (purple). **b** Shows absolute numbers of PQSs in different superfamilies with respect to LTRs and out of LTR regions (INT). **c** Proportion of PQS found within superfamiles present in the LTR region (e.g. out of all PQS+ found in Copia superfamily 64% are located in LTRs). **d** Density of predicted PQS normalized per length in LTR regions, out of LTR regions (INT) and whole elements with respect to main superfamilies

We searched for maize LTR retrotransposons having potential quadruplex-forming sequences (PQS) using *pqsfinder* (Fig. 1; Additional files 3 and 4). We found that about 37% of all families contained at least one PQS (Fig. 1a) with a tendency to have a higher number of PQS in the same element - on average more than 3 PQS per family. LTRs and their immediate neighborhood (less than 350 bp from the end of detected LTR) contain overall fewer PQS than non-LTR regions, what is caused by the shorter length of the LTRs. If the length is considered LTRs show on average more than twice higher density of PQS (per family and kb) than the other regions of the elements. This is even more pronounced in Copia superfamily since the PQS density is more than three times higher in LTRs (Fig. 1d). This also indicates that LTRs are enriched for G4 motifs compared to other regions of the elements.

Surprisingly, the majority (79%) of all high-scoring PQSs in maize elements were accumulated in the minus strand (Fig. 1b). The prevalence of PQS in the minus strand was also seen in Copia LTR retrotransposons but these elements tend to harbour PQS in the plus strand particularly inside LTRs (Fig. 1c). It suggests that if a PQS is located in plus strand of a Copia element then it is preferentially located within the LTRs. Notably in Gypsy retrotransposons it is evident that while 5′-LTRs tend to contain more PQS in the minus strand, 3′-LTRs contain more PQS in the plus strand, with a small peak on the opposite strand present in the immediate vicinity, presumably in the untranslated region (UTR; Additional file 3).

Although LTR retrotransposons tend to harbour more than one PQS in their LTRs, for clarity and convenience we selected 10 elements possessing only one PQS within their LTRs. Since even sequences with very long central loop can form G4s, our selection included five elements with PQS having short loops (up to 8 nucleotides) and five elements with PQS possessing a central loop of 27–49 nucleotides (Additional file 2).

**Fig. 2:**
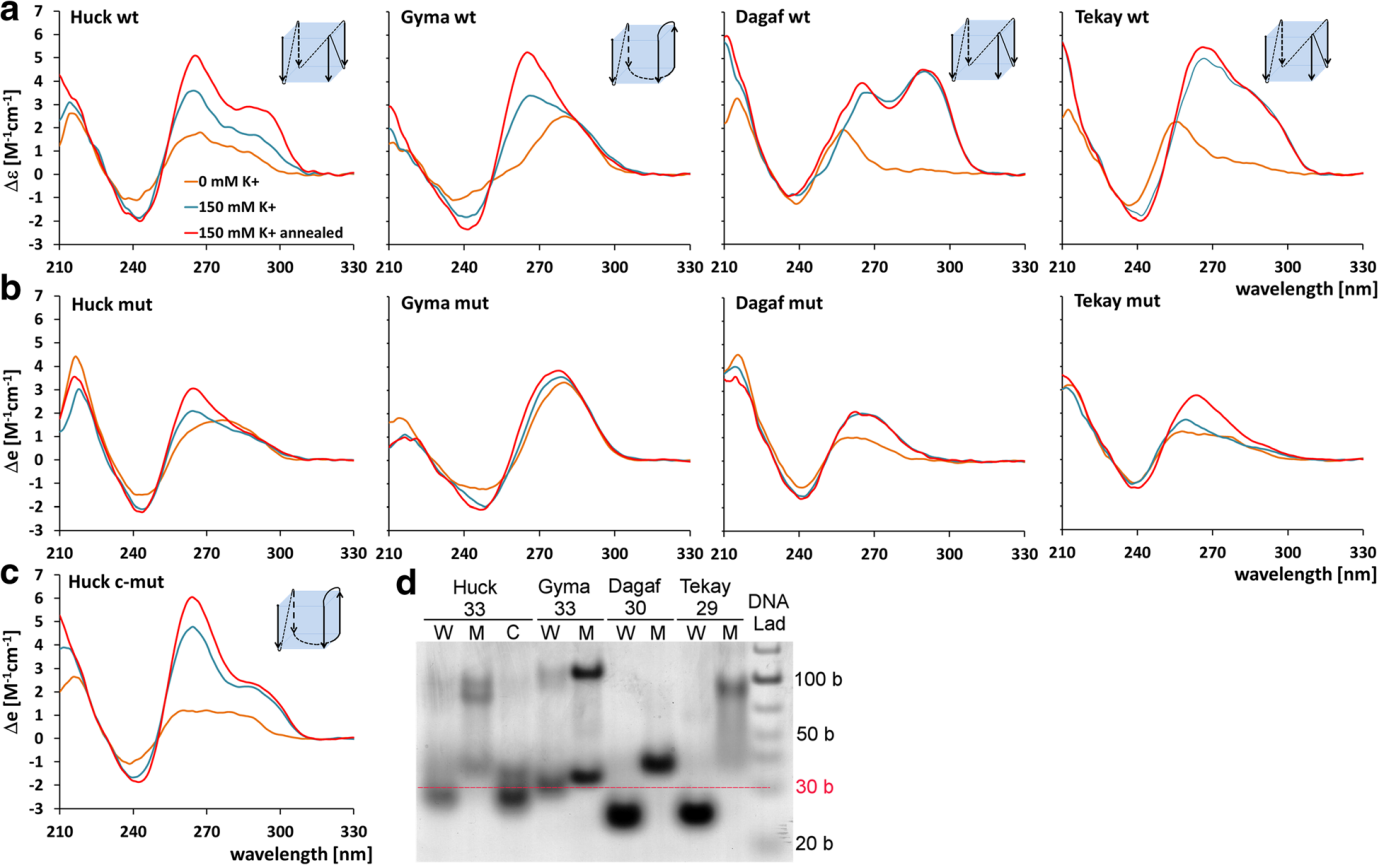
CD spectra of selected oligonucleotides representing the parts of LTRs with wild-type and mutant PQS. **a** CD spectra of oligonucleotides representing wild-type PQS within LTRs from various LTR retrotransposons obtained at different concentrations of potassium ions. The peak at 265 nm corresponds to a parallel-stranded quadruplex. Sketches correspond to the most probable folding of the dominating quadruplex structure according to CD and electrophoretic results. **b** CD spectra of oligonucleotides representing mutant PQS within LTRs of various LTR retrotransposons. **c** CD spectra of Huck LTR retrotransposon having a control CG to TC substitution in G4 loop. **d** Native gel electrophoresis of oligonucleotides in the presence of 150 mM KCl at 28 °C, length of oligonucleotides is indicated below names

To confirm the ability of selected PQS to adopt G4 structures in vitro we measured circular dichroism (CD) spectra using synthetic oligonucleotides (Fig. 2a). We performed UV melting analysis for short loop G4 motifs to determine Tm and to confirm the results obtained by CD (in all cases UV melting was in agreement), and also on oligonucleotides with long loops as they are difficult to assess for G4 formation by CD measurement. Out of five tested oligonucleotides with short loops, four formed G4 in vitro (Table 1) - one oligonucleotide corresponding to the Gyma Gypsy LTR retrotransposon formed a parallel stranded quadruplex as indicated by a high peak at 265 nm. The other three oligonucleotides corresponding to the Huck, Tekay and Dagaf Gypsy LTR retrotransposons formed a 3 + 1 arrangement as indicated by a high peak at 265 nm and a secondary peak at 290 nm (Fig. 2a). Tm values varied from 55 to 62 °C. Six oligonucleotides did not form G4s under the tested conditions (Additional file 5), five of them having long loop and one having short loop PQS.

**Table 1:** Wild-type G4 motifs, their stability and conformation

Element family name G4 motif sequence	T_m_ [°C]	Molecularity	Max delta epsilon	Strand orientation
Huck	62.2	mono	265.5	paral + anti
C*GGGG*ATATA*GGGGG*AACGCA*GGG*ACGC*GGGG*C				
Gyma	56.3	mono	265	paral
CT*GGG*C*GGGG*ATGACGC*GGGG*TAGAGCA*GGG*CT				
Tekay	61	mono	266	paral + anti
CA*GGGG*TTA*GGG*TTA*GGGG*ATTTT*GGG*CC				
Dagaf	55	mono	265	paral + anti
TT*GGG*TGACCTA*GGGG*TA*GGG*TTAA*GGG*AG				

G-runs that potentially participate in G4 formation are written in italics

The ability of tested oligonucleotides to form quadruplexes was also confirmed by native PAGE providing information on molecularity (Fig. 2d). All oligonucleotides formed monomolecular G4s at 27 °C since these migrated faster (they are more compact) than oligonucleotides of the same length.

We tested the effect of mutations on G4s formation by substituting some guanines with adenines with the aim to disrupt G4 formation. The substitutions were carried out on two inner runs of guanines, since we had previously observed that this had a greater effect on G4 formation than in outer G runs ([11], Additional file 2). Our CD spectra measurements as well as native PAGE confirmed that these mutations did indeed disrupt G4 formation (Fig. 2b). For yeast in vivo experiments we chose G4 disruption by mutations rather than stabilization by ligands because (i) the G4s with ligands could behave differently from “ligand-free” G4s and (ii) ligands have large-scale biological effects that could lead to artefacts. The control substitution we introduced in the loop of the Huck G4 sequence verified that the effect was not sequence-specific but correlated with G4 structure as it did not disrupt G4 formation (Fig. 2c).

### Effect of G4 formation on the expression of the lacZ reporter gene in yeast and the detrimental effect of mutations on G4 formation

**Fig. 3:**
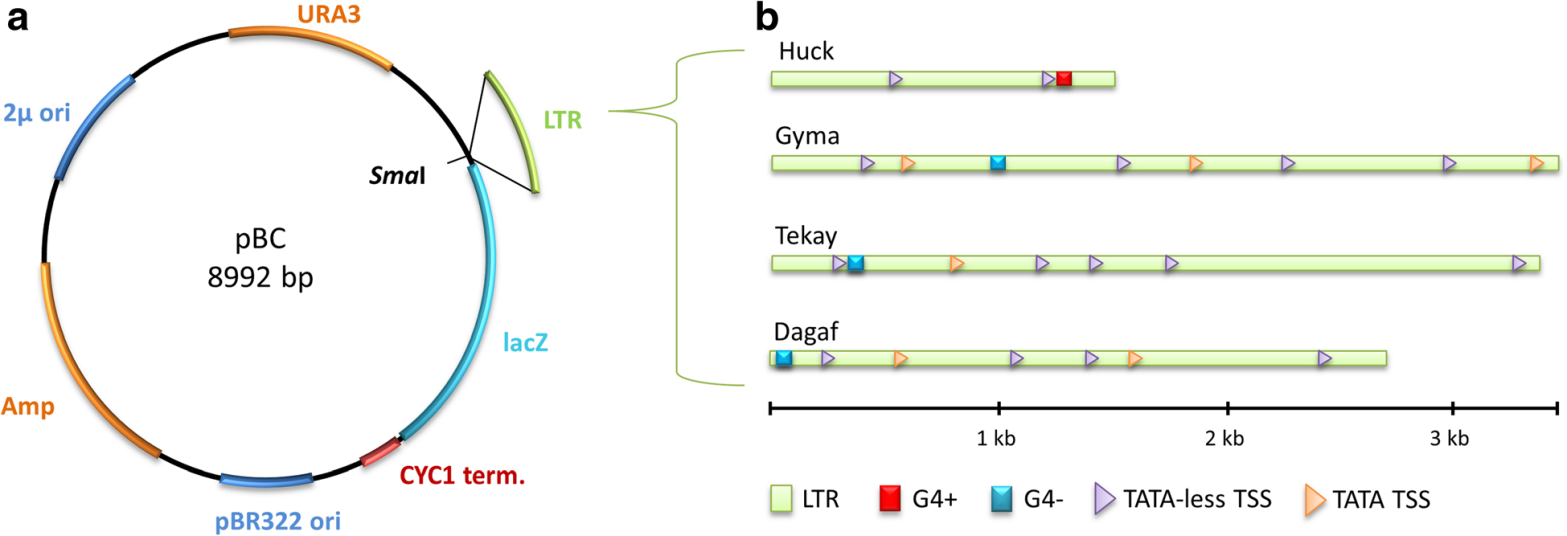
Scheme of plasmid constructs possessing LTRs with PQS. **a** Scheme of pBC in which LTRs (green) were cloned into *Sma*I restriction site. **b** Overview of cloned LTRs. Length is shown on the bottom scale. G4 position and orientation is indicated by red (plus strand) and blue (minus strand) rectangles. Predicted transcriptional start sites (TSS) are also shown, both with TATA box (orange) and TATA-less TSS (purple triangles)

The in vitro CD measurements of short oligonucleotides possessing PQS were followed by an in vivo study of G4 formation contained within longer LTR sequences and its effect on downstream located reporter gene. We cloned selected LTRs amplified from BAC clones upstream of the lacZ reporter gene to create a plasmid construct (Fig. 3a) which was used to transform *Saccharomyces cerevisiae* (CM100). LTRs originated from four LTR retrotransposons: the Huck, Gyma, Dagaf and Tekay families belonging to a Gypsy superfamily and were 1.3–3.5 kb long (Fig. 3b). Gyma, Dagaf and Tekay harboured the G4 motifs on the minus strand closer to the 5′ end of the LTR whereas in the Huck element the G4 motif was situated near the 3′ end of the LTR and was located on the plus strand.

Next we used site-directed mutagenesis on G4 motifs to produce the same PQS mutations as in the CD measurement. The constructs with mutated PQS were used for yeast transformation. Then we compared the LTR driven lacZ expression of wild-type and mutant LTRs in vivo on both protein and mRNA levels.

**Fig. 4:**
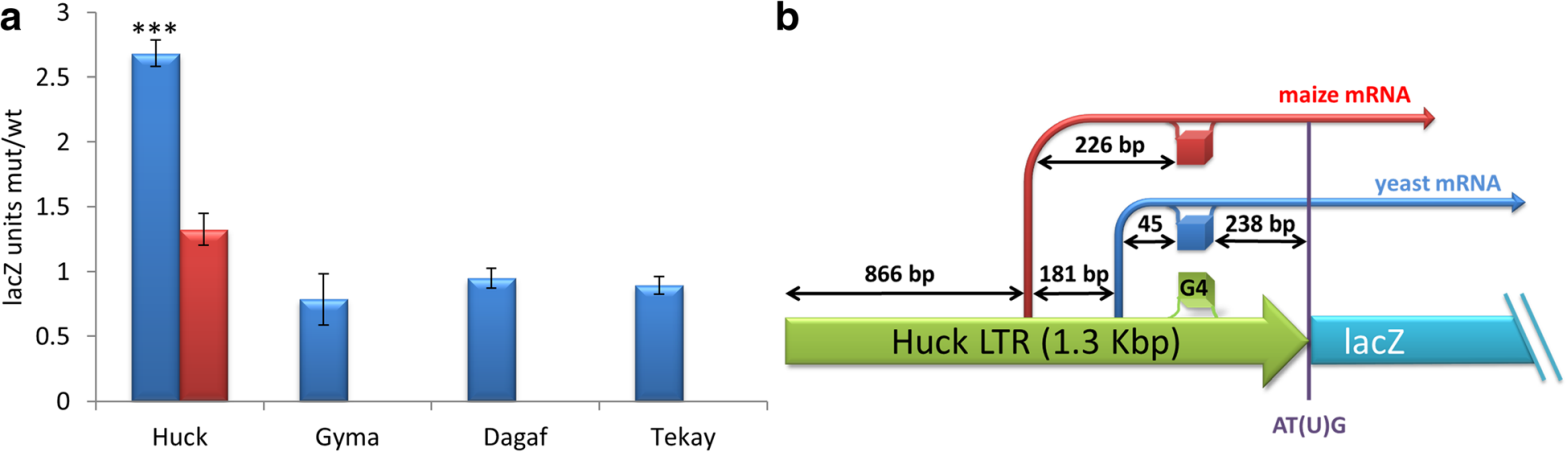
Effect of G4 on expression of lacZ gene in yeast. **a** Comparison of protein expression of lacZ reporter gene cloned downstream of LTR with mutated PQS and wild-type PQS. Red column is comparison of control mutation against wild-type. **b** Transcription start sites (TSS) determined by RACE. Both yeast and maize TSSs located upstream of G4 sequence are shown by blue and red arrows, respectively. The G4 sequence in DNA plus strand (green cube) is transcribed into mRNA (blue or red cubes)

All tested constructs exhibited low lacZ protein levels under the LTR control, the highest expression was observed in the LTR of the Dagaf element that reached up to 20 lacZ units. In three constructs (Gyma, Dagaf and Tekay) lacZ expression was not affected by G4 disruption while in the Huck element the lacZ protein level was more than twice the amount in G4 mutants than in the wild-type and control mutant LTRs (mutation in G4 motif loop) that both harbored stable G4s (*p* < 0,001; Fig. 4a). Contrastingly, there was no difference between wild-type and control mutant LTRs. However, it remained to be determined whether DNA or RNA quadruplex affects lacZ expression.

### Effect of G4 on transcription and the mapping of transcription start sites by RACE

We isolated RNA and performed qRT-PCR in order to assess the effect of G4 formation on transcription and/or translation. We used a URA marker as a reference gene, which was also located on the plasmid construct. No differences were observed in lacZ mRNA levels between wild-type and mutant LTRs. Increases in lacZ protein levels in mutants disrupting G4s inside Huck LTRs in contrast with unaffected levels of mRNA suggest that G4 hampered translation rather than transcription and that quadruplex formation occurs at the RNA level.

In order to determine whether transcription is specific for LTR retrotransposons i.e. being initiated at a promoter located within LTR, and is not a result of read-through (co-transcription), we estimated transcription start sites (TSS) using the Strawberry TSSPlant prediction tool and then performed Rapid Amplification of cDNA Ends (RACE) on both yeast and maize total RNA. We found that the transcription start site of the Huck element is located within the LTR and upstream of the G4 sequence both in yeast and maize although the position of specific TSS differed slightly (Fig. 4b). Notably, the yeast experimentally determined TSS by RACE was in the same position as the one predicted by TSSPlant.

### Stabilization of quadruplexes in maize seedlings grown in the presence of G4-stabilizing ligand NMM and the effect of NMM on LTR retrotransposon expression

**Fig. 5:**
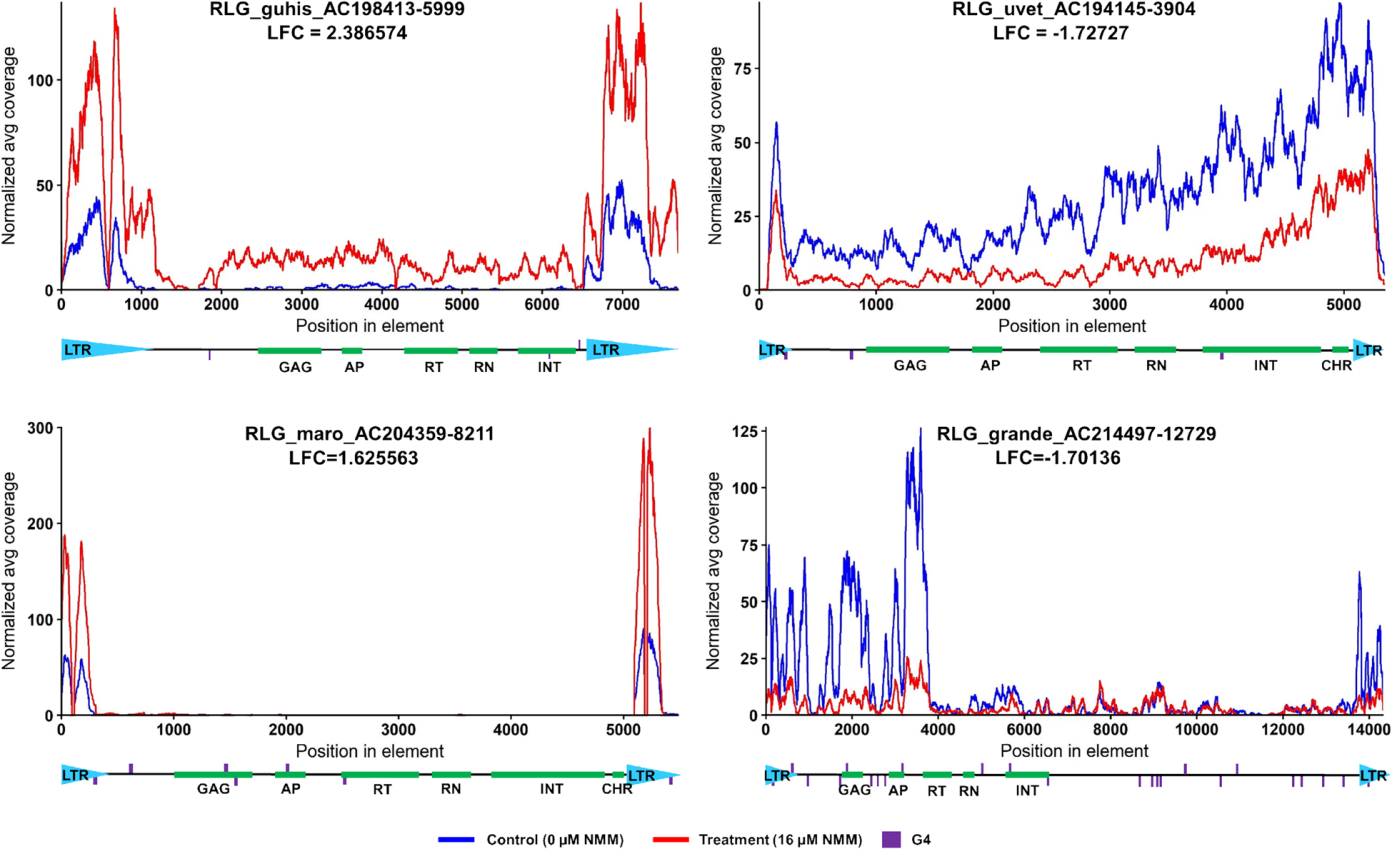
Effect of NMM on transcription of LTR retrotransposons in maize seedlings. Graph of coverage of LTR retrotransposon families by RNA-Seq reads obtained from plants treated with G4 stabilizing drug NMM (red) and from control plants not treated with NMM (blue). Positions of G4 motifs are displayed by purple ticks, G4 motifs on plus strand are above the element, G4 motifs on minus are below the element

In yeast we used mutations of PQS and tested the effect of G4 formation on a very limited number of elements, however, the potential effect of G4 on gene expression in vivo can also be studied by using a G4-stabilizing ligand. Therefore, to know more about the genome-wide G4 stabilization effect on retrotransposons transcription, maize seedlings were grown in the presence of the NMM ligand and polyA RNA sequencing was performed using Illumina. The subsequent analysis of RNA-Seq data revealed that the elements studied above had low transcription and were not differentially expressed. On the other hand, several LTR retrotransposons showed high transcription and were differentially transcribed in the presence/absence of NMM. The Gypsy retrotransposons of Grande and Uvet showed lower transcription in the presence of NMM while in the Guhis and Maro families NMM had stimulatory effect on transcription (Fig. 5).

## Discussion

In this study we showed that the G4 motif, previously confirmed to adopt quadruplex conformation in vitro, located downstream of TSS within the long terminal repeat of LTR retrotransposons, affects the LTR driven expression of the lacZ reporter gene by regulating translation. The translation repression by G4s located in the 5’UTR of the firefly luciferase reporter gene has been well-documented in both cell-free and *in cellulo* systems [35, 36]. Our work belongs to several rare studies, emerging only during recent years, determinating the biological role of quadruplexes in vivo and indicating the importance of non-B DNA conformation in the life cycle of LTR retrotransposons.

Our work on prediction of G4 motifs, revealed that central loop length is an important determinant of in vivo G4 formation. Four out of five tested oligonucleotides with shorter loops readily formed G4s in vitro. Contrastingly, the motifs with longer central loops (27–49 nt) did not readily adopt quadruplex conformation under tested conditions and G4 formation was rather an exception here. Although our study was focused only on the maize LTR retrotransposons, our results are in agreement with previous analyses from 21 plant species that revealed enrichment of G4 motifs within the LTRs of retrotransposons [11]. The difference in PQS number and location (on plus or minus strands) in Copia and Gypsy retrotransposons may be connected with differences in their regulation, mode of amplification and/or the age of families where younger families have more PQS than older ones [11, 12].

The prevalence of PQS in the minus strand suggests that there is selection pressure against the presence of G4 in the plus strand where G4s inhibit the translation and subsequent amplification of retrotransposons. This is consistent with our results showing that the translation of the Huck retrotransposon (possessing G4 in the plus strand) was inhibited while the translation of the Gyma, Tekay and Dagas retrotransposons (possessing a G4 motif in the minus strand) was not affected. Strand specificity in G4-affected processes has also been observed in other systems and organisms. For example, Smestad and Maher [37] demonstrated strand differences in PQS presence in human genes differentially-transcribed in Bloom Syndrome and Werner Syndrome, two disorders resulting in the loss of PQS-interacting RecQ helicases.

Although we demonstrated the effect of the G4-stabilizing drug NMM on the transcription of LTR retrotransposons, irrespective of their subsequent impact on translation, the elucidation of the role of G4s in transcription and other steps of the LTR retrotransposon life-cycle needs further research. It remains a question to what extent does the positive or negative effect of G4 on transcription depend on the LTR retrotransposon family and its mode of regulation. Moreover, when assessing the differences between the G4 effect on transcription and translation in yeast and maize, we should keep in mind that there are different cellular factors binding the G4s in each case.

The inhibitory or stimulatory effect of G4s on LTR retrotransposons expression can also be explained by the formation of quadruplex structures within only a specific genomic context and/or in particular cellular (ionic and protein) environments. Such an explanation is consistent with the finding that quadruplexes are globally unfolded in eukaryotic cells [8]. The abundance and strand-location (plus or minus) of G4 motifs within retrotransposons is probably the result of an interplay between the propensity of mobile elements to amplify and the demand of the cell to suppress retrotransposon activity in order to maintain genome and cell integrity.

We have demonstrated the effect of G4s on the transcription of LTR retrotransposons in maize and on their translation in yeast but we cannot exclude that G4s affect also other steps of LTR retrotransposon life cycle. The effect of G4 on other life cycles has previously been shown in closely related retroviruses, e.g. in HIV-1 nucleocapsid proteins are bound to the G4 structure of the preintegration genome leading to the initiation of the virion assembly [38]. In addition, sequences near the central polypurine tract that form bi-molecular quadruplex also facilitate strand transfer and promote template switching during reverse transcription of HIV-1 [39, 40]. Moreover, the formation of bi-molecular quadruplex is believed to stabilize the pairing of the two RNA genome molecules which ensures the encapsulation of both genome copies in virion [41, 42].

It is also possible that in some cases G4s take part in retrotransposon stress activation. RNA quadruplexes are essential for cap-independent translation initiation [43] during which the 40S subunit of the ribosome is recruited into a position upstream or directly at the initiation codon via a specific internal ribosome entry site (IRES) element located in the 5’UTR. In plants, stress conditions (drought, high salinity and cold) lead to dehydration and thus increase molecular crowding in the cell favouring G4 formation [44]. Furthermore, cap-independent translation is often related to stress states and diseases such as cancer [45] and, remarkably, stress also activates transposable elements that in turn, by inserting their new copies, probably spread new G4 motifs throughout genomes [46]. In this way, quadruplex DNA can participate both in short-term (physiological) and long-term (evolutionarily) responses to stress.

Our finding that all four tested G4s adopted intramolecular (monomolecular) quadruplex agrees with its regulatory role during translation or transcription where a single RNA/DNA molecule participates. Moreover, all our G4s show parallel strand orientation prevalence supporting their potential role during transcription since promoter-associated quadruplexes tend to be parallel-stranded [3].

## Conclusions

Our study provides, to our knowledge, the first experimental evidence that quadruplex DNA located within the long terminal repeat of LTR retrotransposons can affect the expression of plant LTR retrotransposons in vivo: (i) mutation disrupting G4 in the LTR resulted in a higher translation level of a downstream located reporter gene in yeast compared to the wild-type the G4 motif and (ii) the G4 stabilizing drug NMM affected transcription of LTR retrotransposons in maize. This demonstrates that quadruplex DNA plays a regulatory role in the maize LTR retrotransposon life-cycle. Therefore, stabilization of quadruplexes present in LTR retrotransposons under specific cellular conditions can, thanks to the multicopy character of LTR retrotransposons, influence whole genome dynamics as well as represent the abundant barriers for DNA replication.

## Additional files

Additional file 1: Annotation of all 579 maize TEs included in this study. The presence and position of detectable LTRs, PBS and PPT sequences (LTR Finder), protein-coding domains (BLASTX) and potential quadruplex sequences (PQS; pqsfinder). White rectangles represent LTRs, blue rectangles are common TE domains (labelled) or other domains detected in Uniprot (unlabelled). Small blue bars are PQS with score > 24 (> 64 larger bar). (PDF 927 kb)

Additional file 2: Overview of oligonucleotides, BAC clones and primers used in study. Name is derived from Maize TE Database. BAC referes to ZMMBBc library coordinates of clones containing given element. G4 motif coresponds to oligonucleotides used for CD measurments and UV melting. Guanine tracks and mutations are highlighted by bold bigger font in wilde types and mutants respectively. Forward and reverse primers were used for element amplification from BAC clones, the product length is indicated as last colum. Mutagenic primers were used for generating constructs with mutatnt LTRs, mutations are highlighted as mentiond above. RACE and RACE nested primers were used for rapid amlification of cDNA ends in yeast and maize. (XLSX 10 kb)

Additional file 3: Occurrence of high-scoring PQS along maize LTR retrotransposons. The distribution of PQS containing a minimum of four adequately spaced G runs in the sense strand (PQS3+, upper row) and antisense strand (PQS3-, lower row) as identified by *pqsfinder* where **(a)** score > 64 and **(b)** score > 25. Gypsy (RLG), Copia (RLC) and other (RLX) superfamilies are shown in separate columns. Frequency (vertical axis) represents the number of PQS present in a window covering 2% of TE length. 75% of LTRs fall within the black rectangles shown below the horizontal axis (3rd quartile = 0.125; mean LTR length = 0.100; maximum length = 0.427). (TIFF 3702 kb)

Additional file 4: Overview of families, PQS and average lengths. **Table S1.** Families possessing PQS score > 64. **Table S2.** Number of PQS score > 64 in superfamilies. **Table S3.** Average lengths of LTRs, non-LTR regios and whole elements. (XLSX 13 kb)

Additional file 5: CD spectra of oligonucleotides without G4-forming ability. CD spectra of oligonucleotides representing wild-type PQS from various LTR retrotransposons obtained at different concentrations of potassium ions (orange: 0 mM K+; blue: 150 mM K+ and red: 150 mM K+ after annealing). Debeh, Nobe, Hooni, Wuwe and Prem1 are oligonucleotides with long middle loop. Flip has short middle loop. (TIFF 883 kb)

## Abbreviations

ANOVA: Analysis of variance
BAC: Bacterial artificial chromosome
CD: Circular dichroism
cDNA: Complementary DNA
CPM: Counts per million
DNA: Deoxyribonucleic acid
G4: G-quadruplex
HSD: Honest significant difference
LFC: Log fold change
LTR: Long terminal repeat
mRNA: Messenger RNA
NMM: N-methylmesoporphyrin IX
PCR: Polymerase chain reaction
PQS: Potential quadruplex-forming sequence
RACE: Rapid amplification of cDNA ends
RNA: Ribonucleic acid
TE: Transposable element
TSS: Transcription start site
UTR: Untranslated region
