# Supplementary material for: Quadruplex DNA in long terminal repeats in maize LTR retrotransposons inhibits the expression of a reporter gene in yeast
Source: BMC Genomics. 2018 Mar 6;19:184. doi: 10.1186/s12864-018-4563-7 (PMC5838962; doi:10.1186/s12864-018-4563-7)

RLC_ajit_AC191382-3096

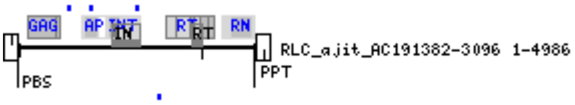

RLC_atop_AC195158-4437

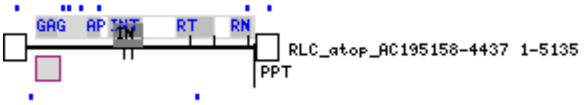

RLG_bavav_AC208118-9781

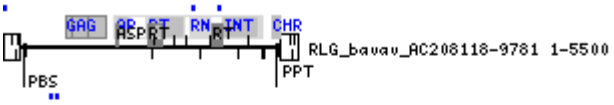

RLG_bomevy_AC195138-4424

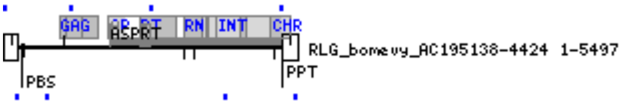

RLC_bote_AC211535-11252

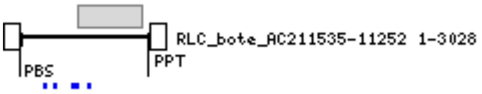

RLG_bowuow_AC212139-11611

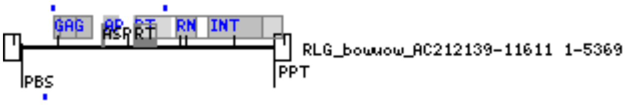

RLG_bygum_AC188125-2053

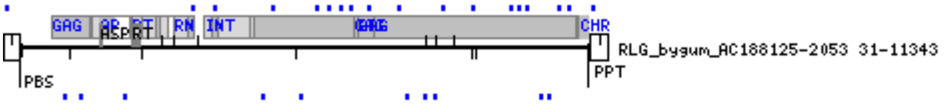

RLC_debeh_AC177840-246

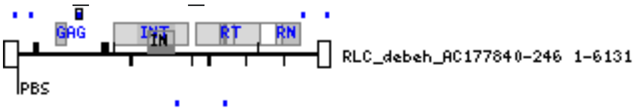

RLC_dijap_AC211466-11198

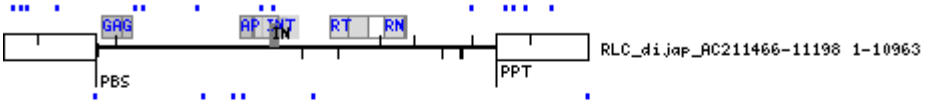

RLC_dolovu_AC189774-2249

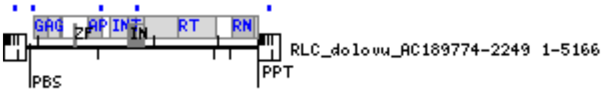

RLC_dolovu_AC211740-11400

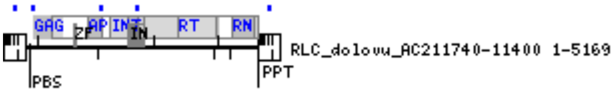

RLC_donuil_AC196196-5026

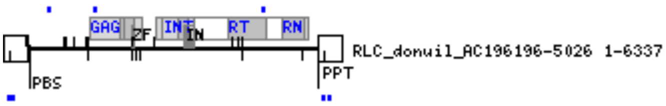

RLC_ehahu_AC202972-7568

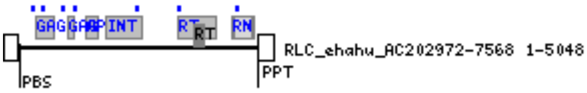

RLG_ekoj_AC194603-4173

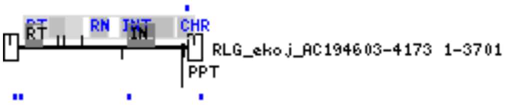

RLC_eninu_AC191055-2893

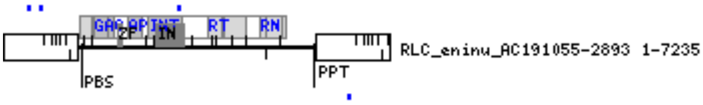

RLC_erev_AC194741-136

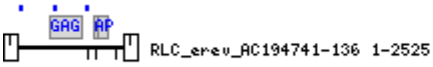

RLG_ewiut_AC194106-3871

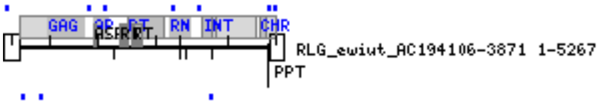

RLC_finaij_AC194312-4026

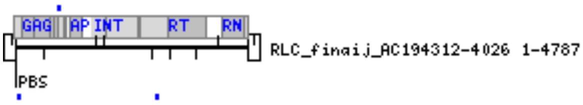

RLC_anar_AC206985-9422

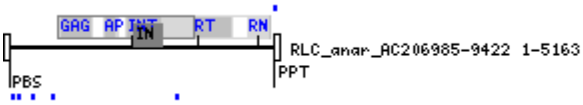

RLC_depuw_AC208349-9837

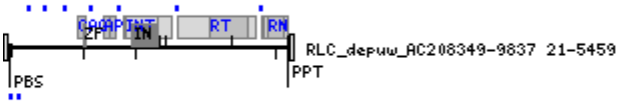

RLC_hago_AC195807-4722

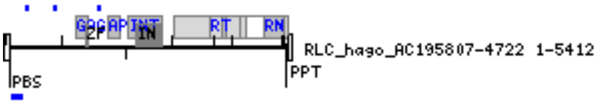

RLC_totu_AC194464-4142

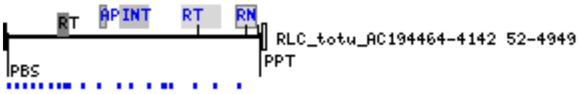

RLC_tufe_AC211502-11228

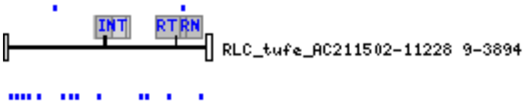

RLC_victim_AC182414-456

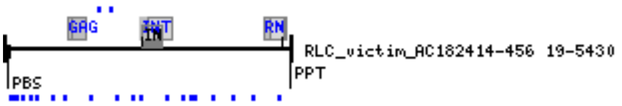

RLC_victim_AC183319-577

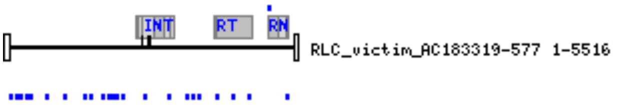

RLC_waneer_AC195414-4606

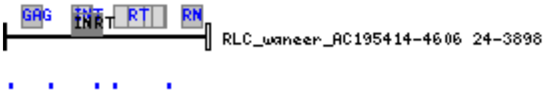

RLC_fipi_AC195215-4479

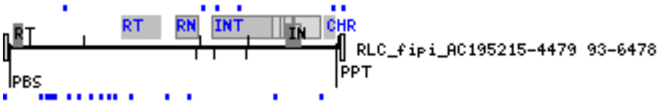

RLC_ajipe_AC183372-580

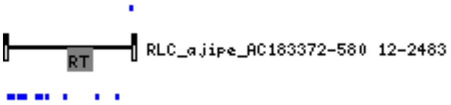

RLX_alaw_AC197914-166

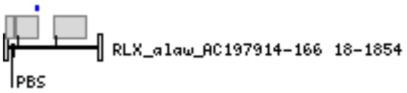

RLC_bihar_AC195875-4766

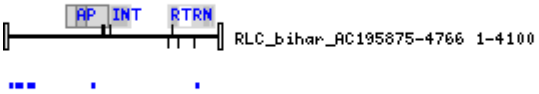

RLX_bula_AC195180-138

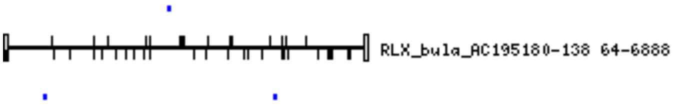

RLX_daju_AC190492-79

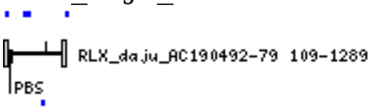

RLC_dugiab_AC207724-9683

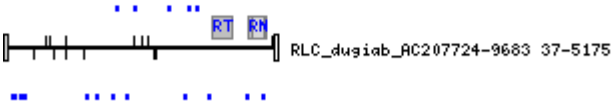

RLC_ebel_AC188777-2128

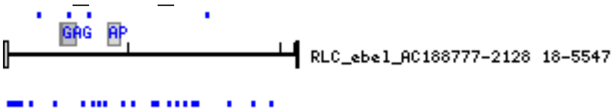

RLX_habu_AC195917-4802

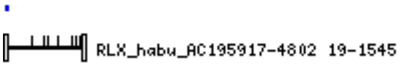

RLX_hiimam_AC207734-9687

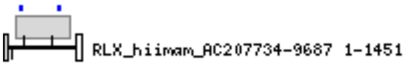

RLX_ilyl_AC196209-163

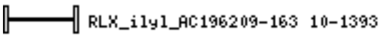

RLC_jelat_AC194217-3960

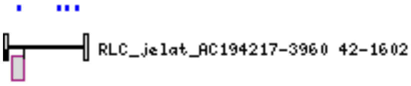

RLX_kaise_AC203928-8059

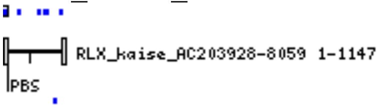

RLX_naseup_AC196428-5108

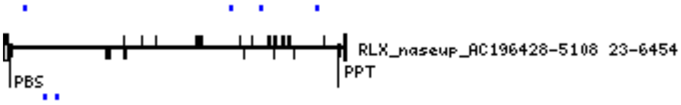

RLX_owume_AC207119-9459

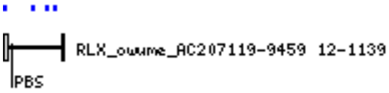

RLX_raga_AC204541-8305

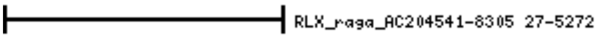

RLX_small_AC195943-142

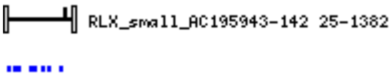

RLX_small_AC217574-13522

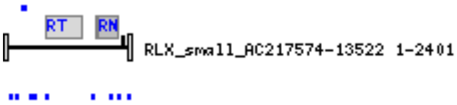

RLC_ubat_AC212211-11652

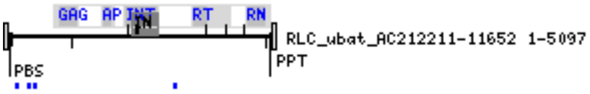

RLX_ugymos_AC182443-149

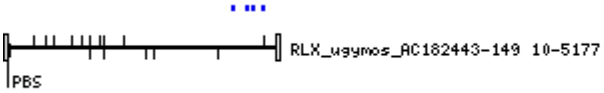

RLC_afad_AC199807-6594

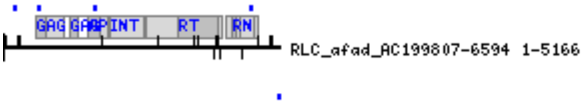

RLG_dadeir_AC201957-7187

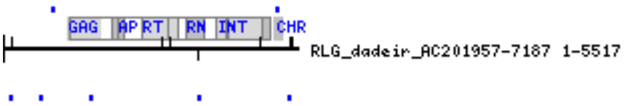

RLC_giepum_AC197531-5634

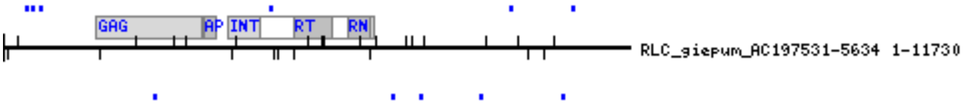

RLG_ikal_AC190798-2599

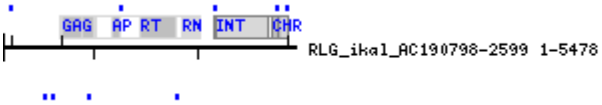

RLG_labu_AC188126-2057

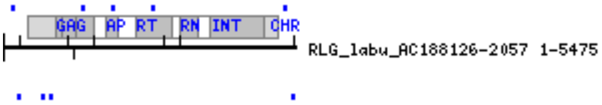

RLG_lise_AC211467-11199

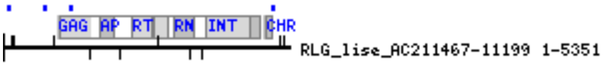

RLG_lute_AC217373-13492

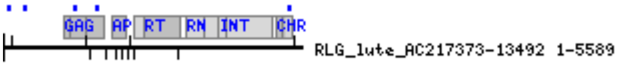

RLX_mijuw_AC190986-101

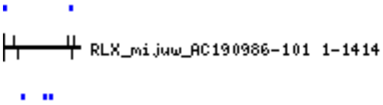

RLC_opie_AC202020-7258

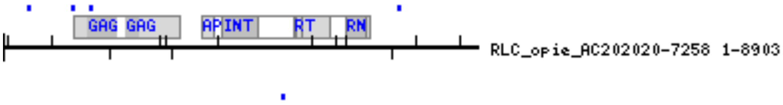

RLC_wawo_AC216857-13424

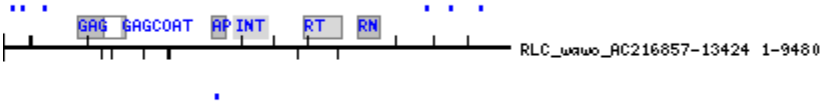

RLG_cinful-zeon_AC186530-1513

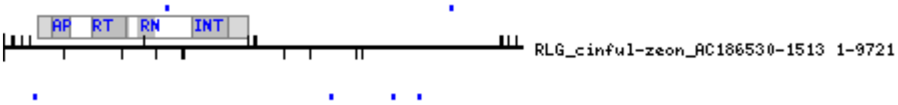

RLG_cinful-zeon_AC201757-7049

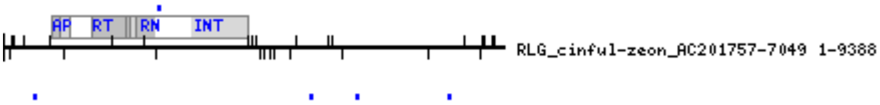

RLG_doke_AC197224-5479

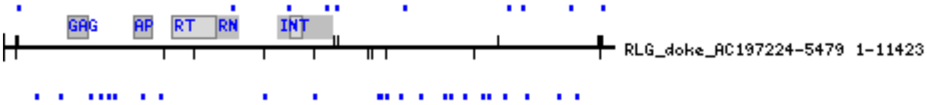

RLG_flip_AC193970-3791

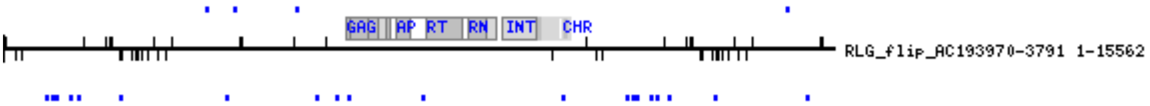

RLG_flip_AC194904-4319

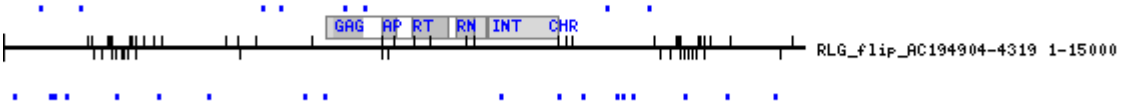

RLG_flip_AC203163-7675

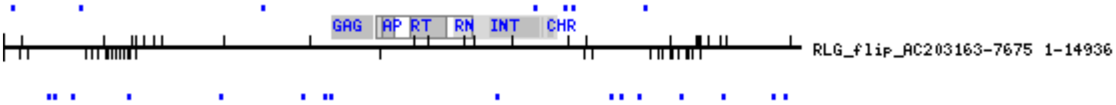

RLG_flip_AC208040-9765

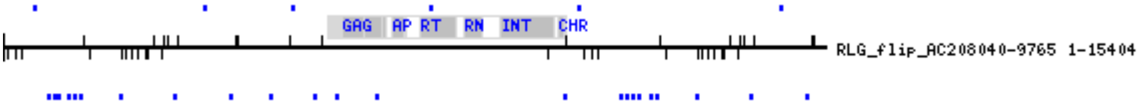

RLG_flip_AC211750-11412

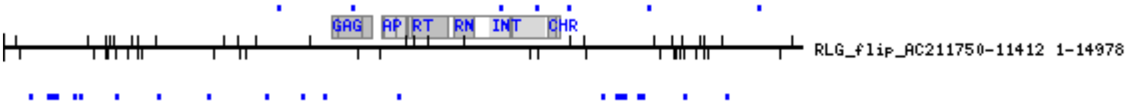

RLG_flip_AC213672-12280

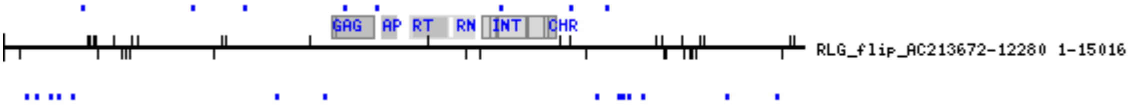

RLG_flip_AC214266-12595

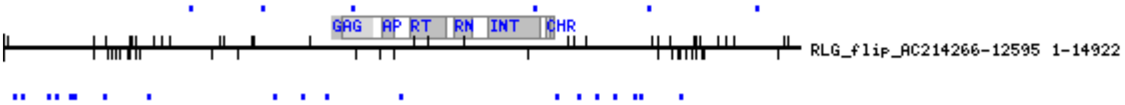

RLG_grande_AC190611-2432

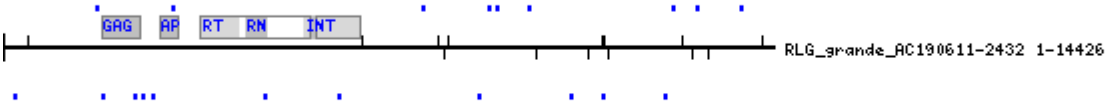

RLG_grande_AC214497-12729

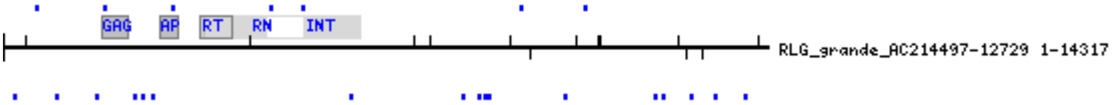

RLG_gyma_AC189750-2238

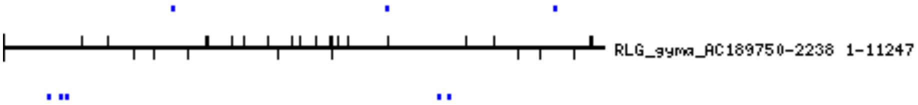

RLG_gyma_AC190530-2365

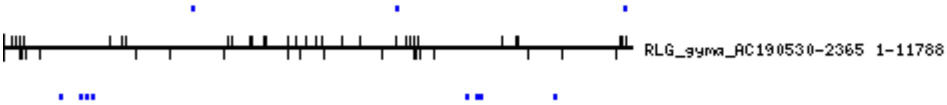

RLG_gyma_AC197250-5506

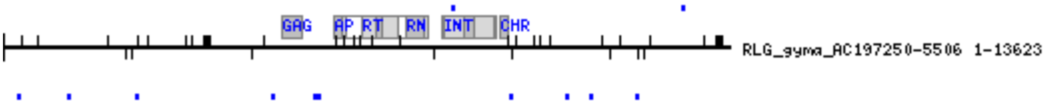

RLG_gyma_AC198939-6228

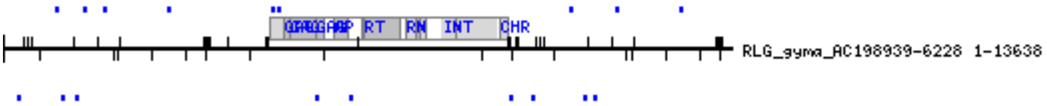

RLG_gyma_AC206653-9288

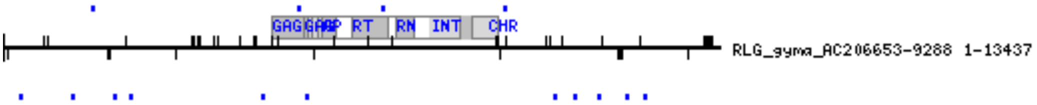

RLG_gyma_AC212146-11616

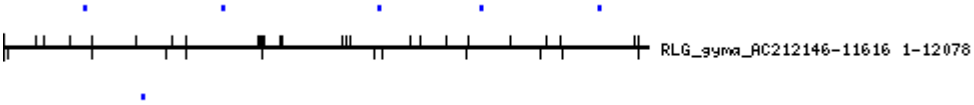

RLG_huck_AC186577-1525

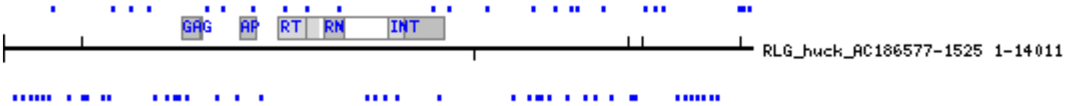

RLG_huck_AC199418-6452

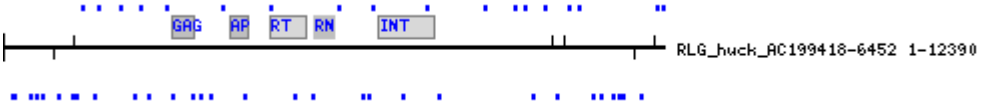

RLG_huck_AC199418-6452

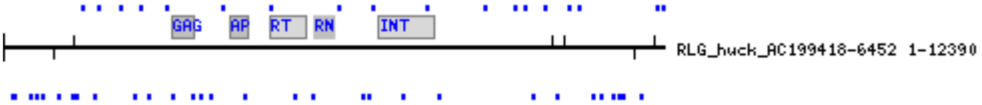

RLG_huck_AC199418-6452

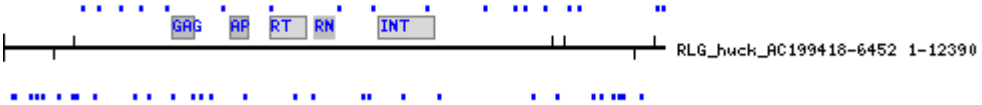

RLG_riiryl_AC193745-3743

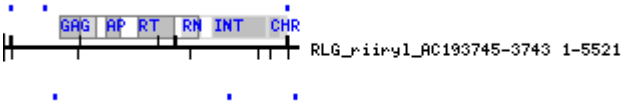

RLG_xilon-diguus_AC185317-1058

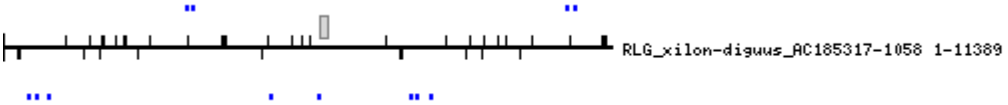

RLX_afeke_AC209700-10302

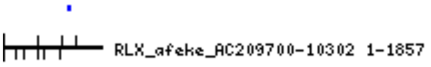

RLX_afuv_AC186332-26

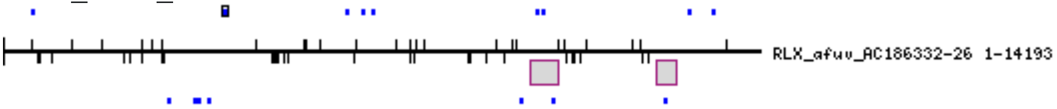

RLX_anim_AC206032-183

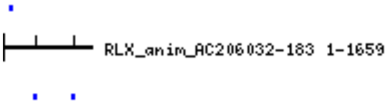

RLX_arar_AC208428-9858

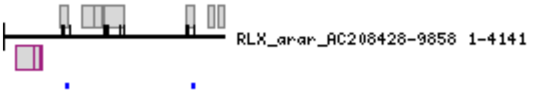

RLX_baso_AC192251-3423

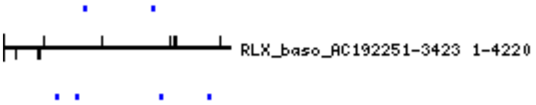

RLX_buire_AC194484-4158

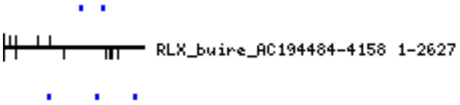

RLC_ebel_AC195143-4427

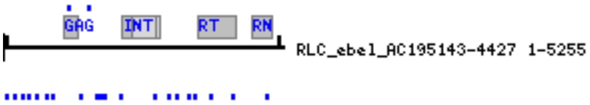

RLX_eguh_AC188982-71

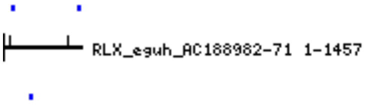

RLX_elalal_AC211887-11443

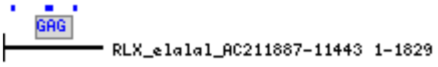

RLX_epom_AC196256-5041

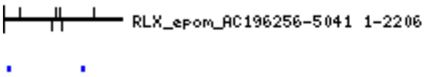

RLX_fosu_AC194180-126

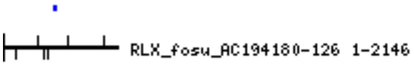

RLX_fosu_AC194180-3925

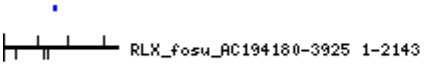

RLX_iwik_AC203371-7824

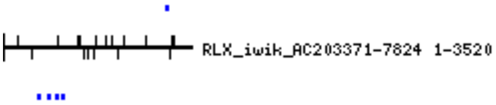

RLX_jakek_AC185468-151

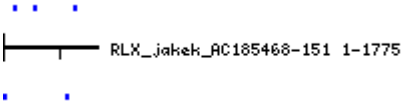

RLX_kahoba_AC186618-29

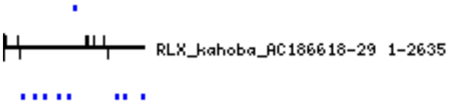

RLX_kahowu_AC205018-178

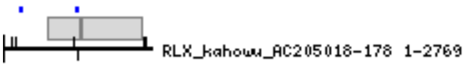

RLX_luteja_AC183974-769

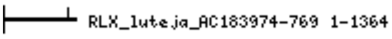

RLX_mewu_AC186332-152

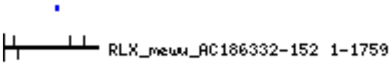

RLG_ojokat_AC204297-8192

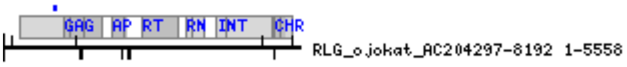

RLX_oviis_AC209739-10346

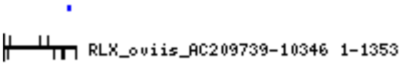

RLG_tekay_AC200856-6996

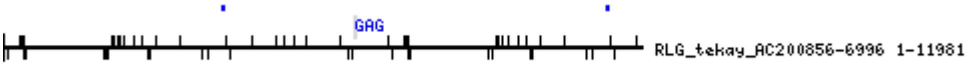

RLX_teuta_AC191650-108

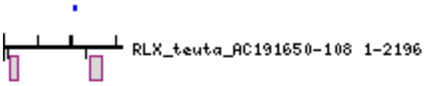

RLX_uluil_AC185306-17

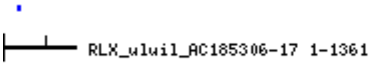

RLX_urogor_AC186334-1406

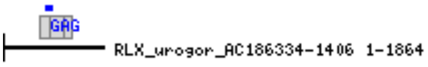

RLG_wuwe_AC191412-107

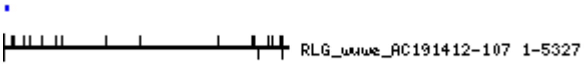

RLX_wuywu_AC190718-2536

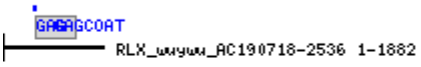

RLC_ypel_AC215810-13168

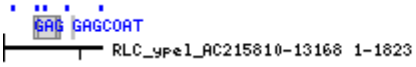

RLC_bovo_AC197547-5647

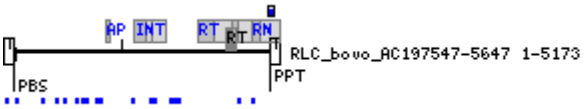

RLG_ewiut_AC194106-3871

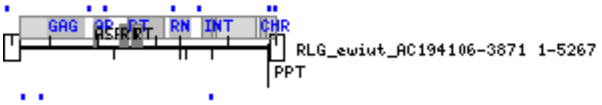

RLG_fuved_AC204055-8151

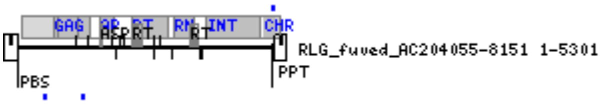

RLC_giepum_AC211155-11010

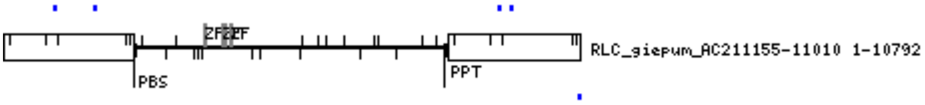

RLC_giepum_AC211251-11074

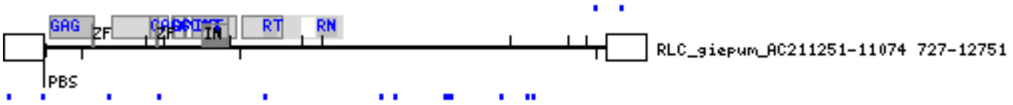

RLC_gudyeg_AC206942-9404

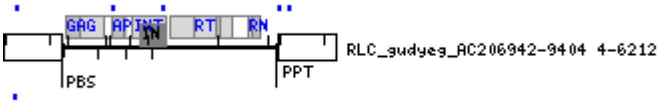

RLC_guvi_AC185473-1128

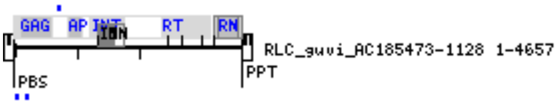

RLG_guwiot_AC207006-9429

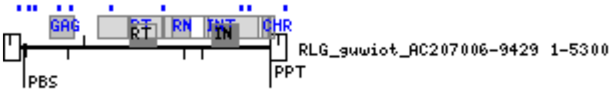

RLG_gylu_AC188833-2139

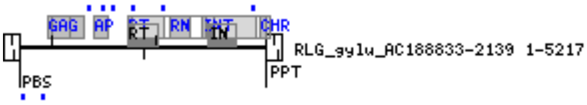

RLC_huta_AC210008-10542

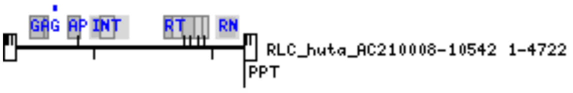

RLG_hute_AC204317-8199

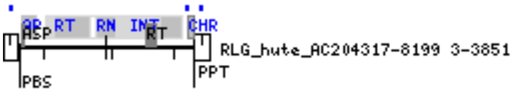

RLC_ibulaf_AC186801-1662

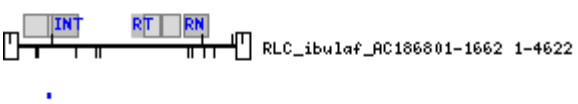

RLG_ijaat_AC199510-6486

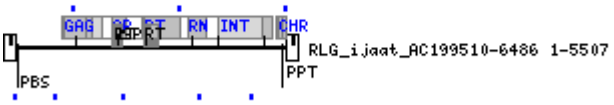

RLC_ijiret_AC195138-4425

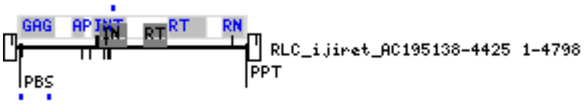

RLC_ijiret_AC211361-11134

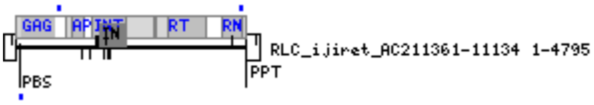

RLG_iwim_AC203300-7761

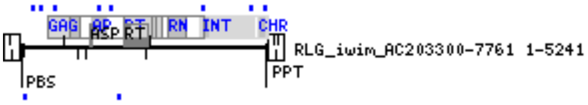

RLC_ji_AC182107-448

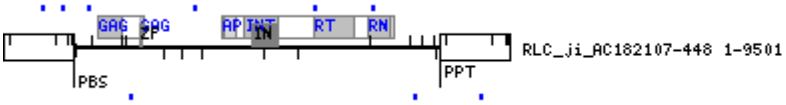

RLC_ji_AC186528-1508

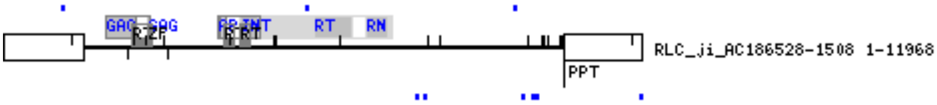

RLC_ji_AC190978-2799

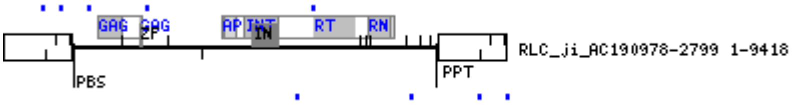

RLC_ji_AC192600-3526

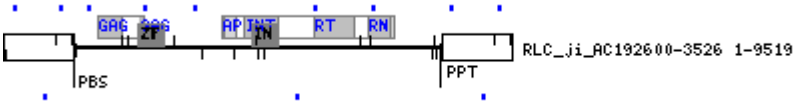

RLC_ji_AC193479-3665

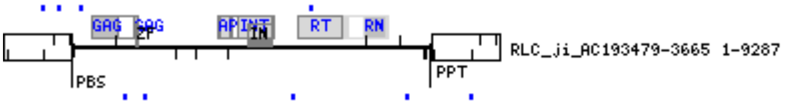

RLC_ji_AC195394-4599

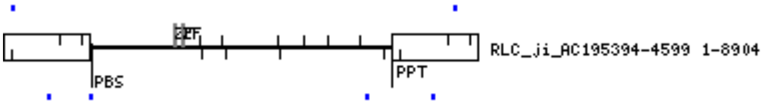

RLC_ji_AC197052-5349

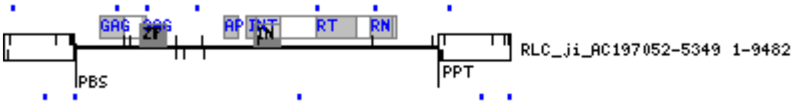

RLC_ji_AC200613-6936

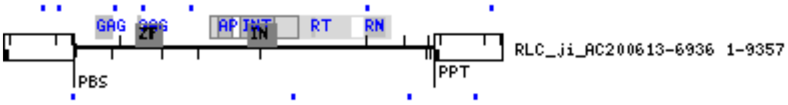

RLC_ji_AC202456-7443

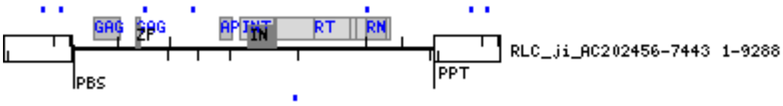

RLC_ji_AC204382-8228

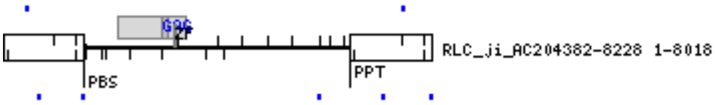

RLC_ji_AC207234-9488

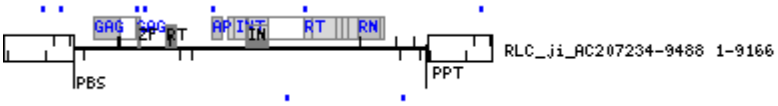

RLC_ji_AC209892-10463

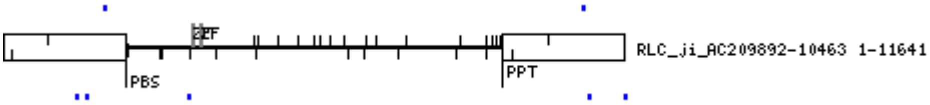

RLC_ji_AC210731-10832

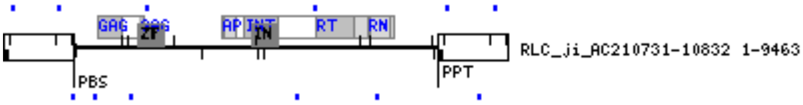

RLC_ji_AC211489-11215

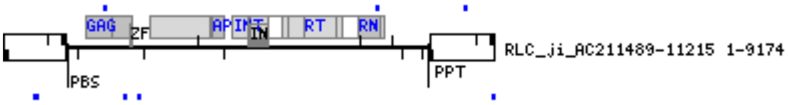

RLC_ji_AC213834-12382

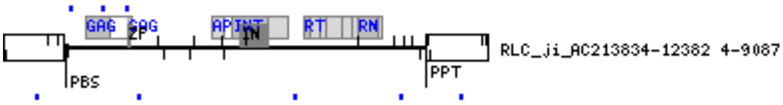

RLC_ji_AC215728-13156

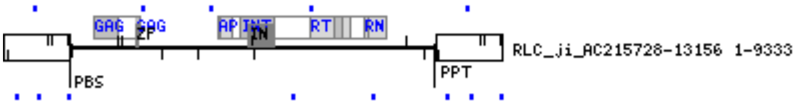

RLG_kase_AC212771-11956

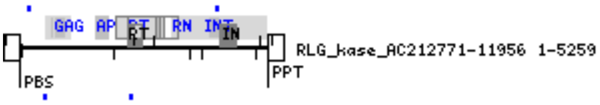

RLG_kise_AC204537-8302

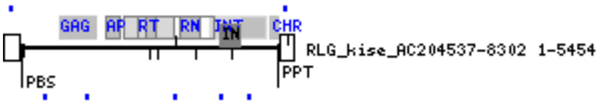

RLX_kupu_AC216069-13264

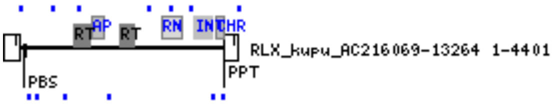

RLC_kuvi_AC207313-9512

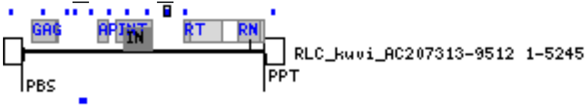

RLC_kuvi_AC207807-9734

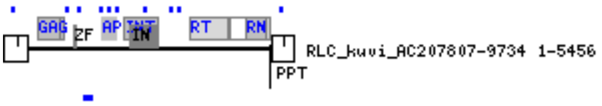

RLC_labe_AC203760-7937

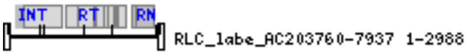

RLG_lowy_AC190530-2366

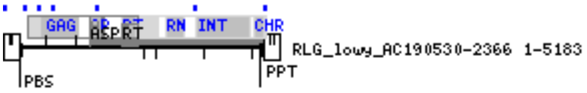

RLC_lusi_AC198175-5903

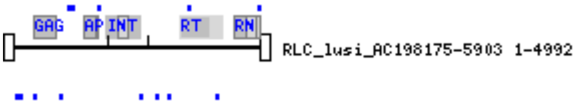

RLG_lute_AC206502-9225

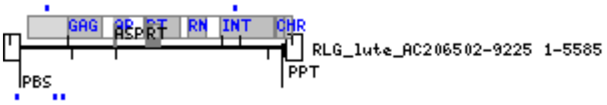

RLG_lywy_AC194967-4386

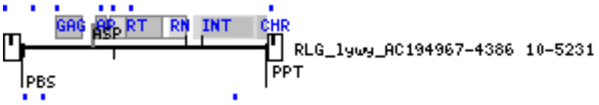

RLC_machiavelli_AC200490-6883

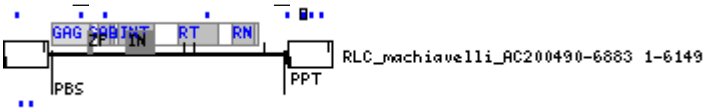

RLC_mauky_AC195954-4843

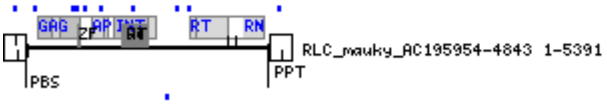

RLG_mywur_AC197359-5559

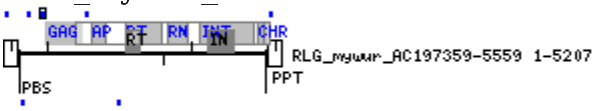

RLC_nene_AC204294-8188

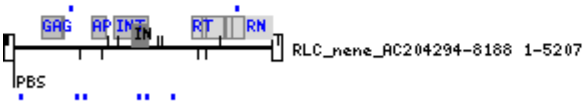

RLC_niki_AC217821-13540

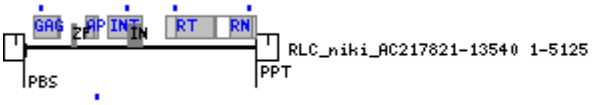

RLG_notu_AC200254-6827

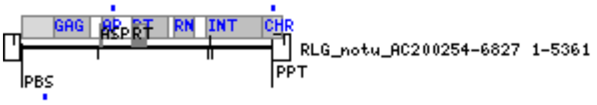

RLC_nowuv_AC215510-13095

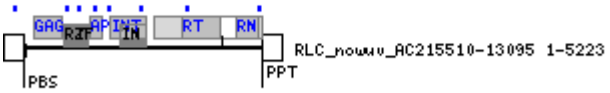

RLG_oguod_AC209724-10327

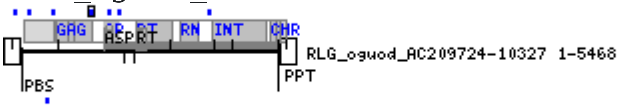

RLG_omoha_AC184843-894

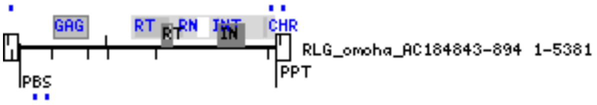

RLC_opie_AC185480-1137

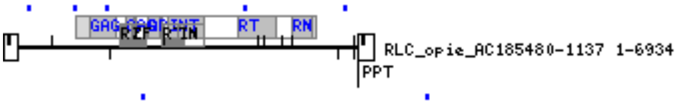

RLC_opie_AC187149-1780

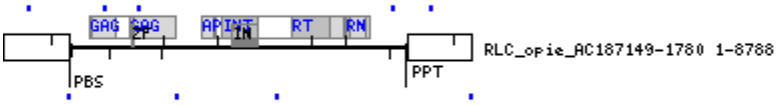

RLC_opie_AC187207-1792

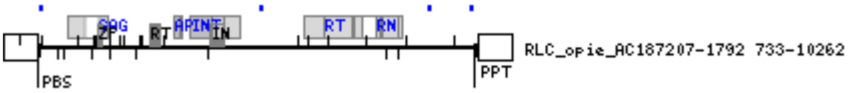

RLC_opie_AC188002-2029

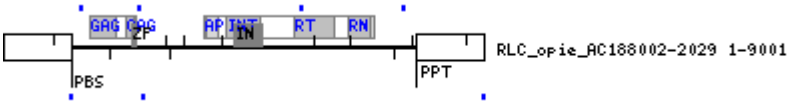

RLC_opie_AC196469-5133

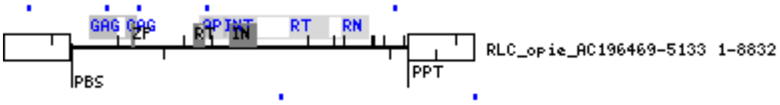

RLC_opie_AC197084-5382

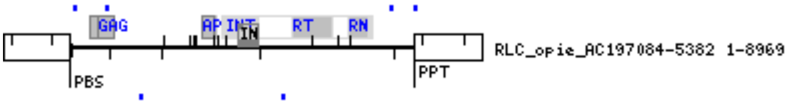

RLC_opie_AC197201-5474

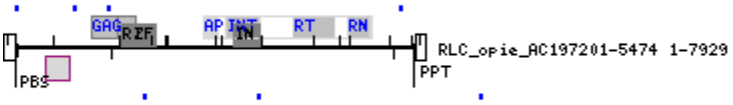

RLC_opie_AC197691-5727

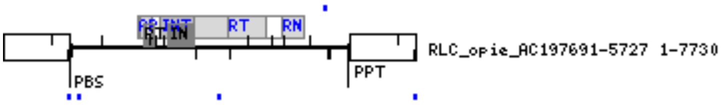

RLC_opie_AC198173-5898

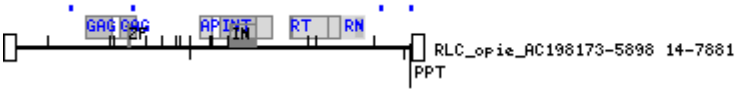

RLC_opie_AC198924-6206

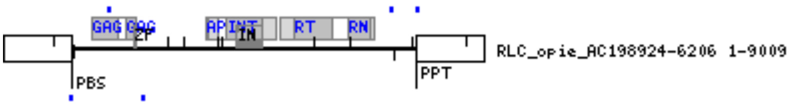

RLC_opie_AC201793-7083

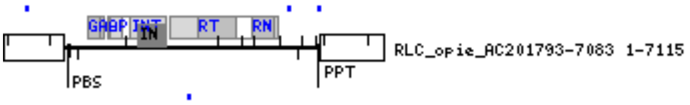

RLC_opie_AC202033-7274

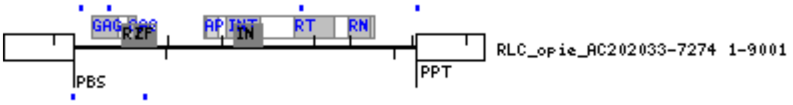

RLC_opie_AC210610-10772

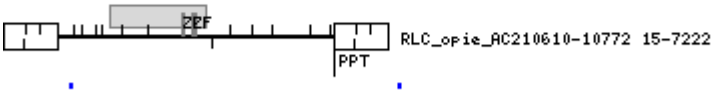

RLC_opie_AC211653-11340

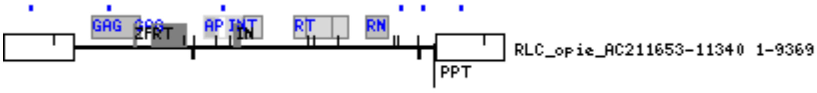

RLC_opie_AC214122-12495

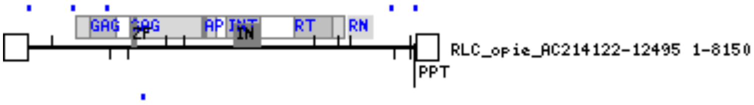

RLC_opie_AC217577-13524

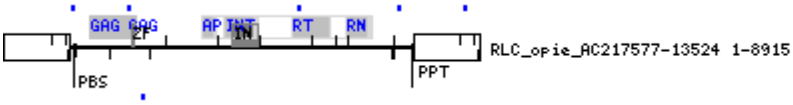

RLG_ovikoh_AC200886-1-999999

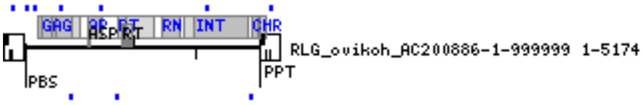

RLC_owiit_AC196151-4998

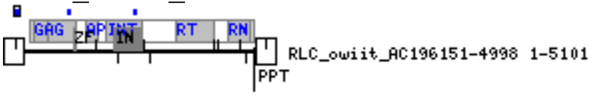

RLC_pifo_AC209023-10096

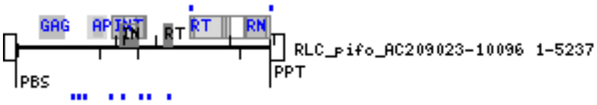

RLC_raider_AC197426-5583

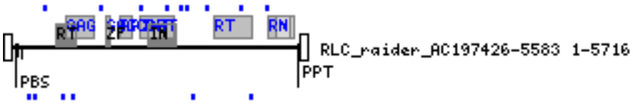

RLC_raider_AC209705-10304

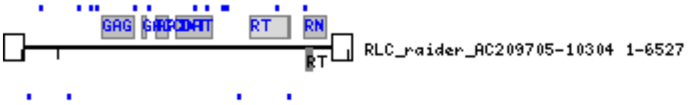

RLC_rely_AC199569-6530

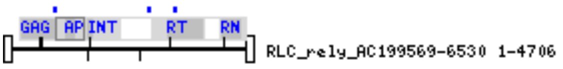

RLX_ruda_AC195952-4839

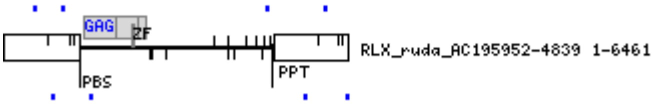

RLX_ruda_AC202870-7495

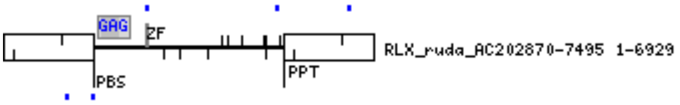

RLX_ruda_AC206281-9164

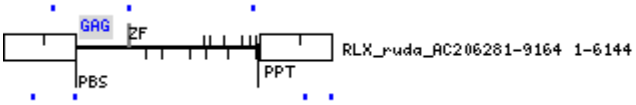

RLG_rufefu_AC193384-3596

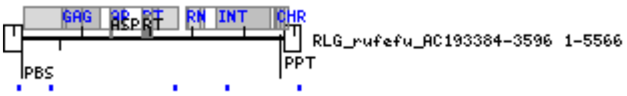

RLC_ruhi_AC205536-8890

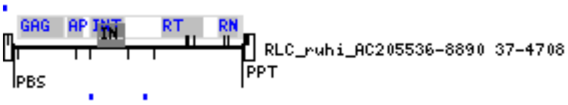

RLG_sawujo_AC193398-3610

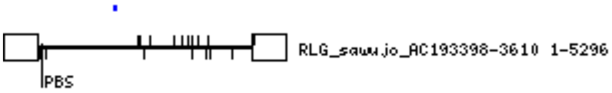

RLC_seufyt_AC194940-4361

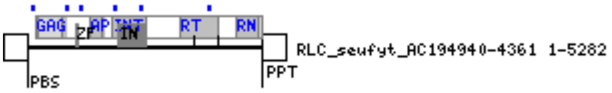

RLG_seuwe_AC210840-10901

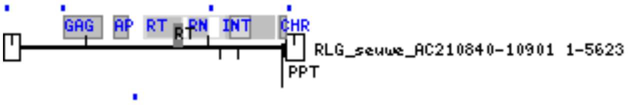

RLG_soger_AC204598-8342

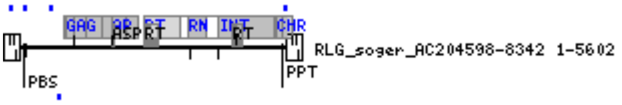

RLG_sowu_AC215679-13136

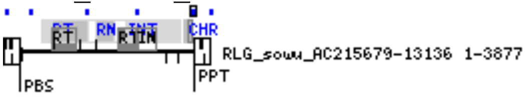

RLC_stonor_AC212476-11849

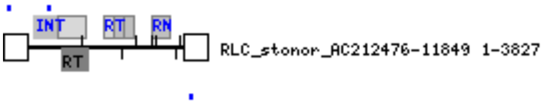

RLC_taname_AC194273-3998

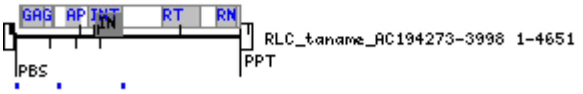

RLC_tata_AC205418-8792

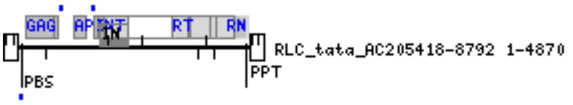

RLC_tatu_AC212076-11563

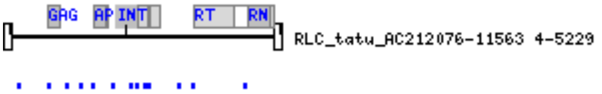

RLX_tisy_AC193518-123

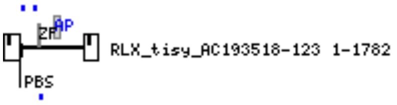

RLC_tiwe_AC187032-1709

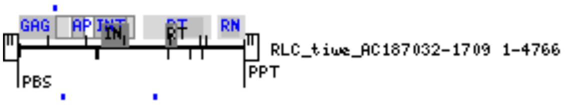

RLC_tiwewi_AC210937-10935

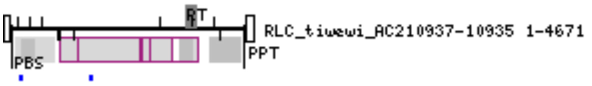

RLC_ubep_AC210005-10539

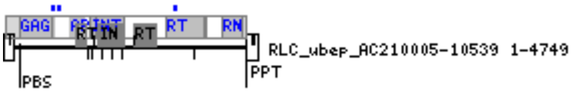

RLG_udav_AC196188-5022

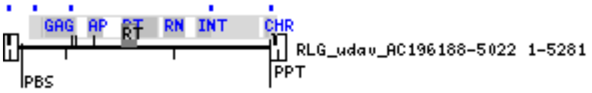

RLG_ufonah_AC194064-3852

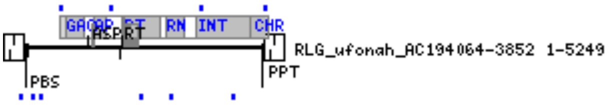

RLC_ugog_AC206863-9375

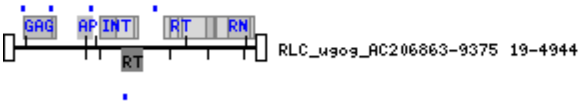

RLC_uhun_AC200619-6943

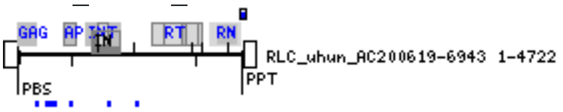

RLG_ulik_AC205416-8790

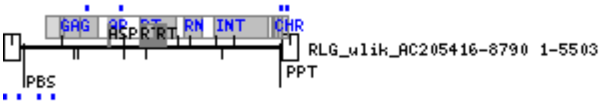

RLC_ulyg_AC196397-5082

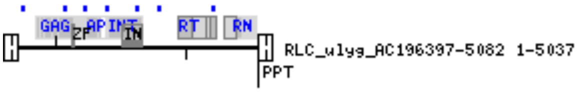

RLG_ures_AC199044-6279

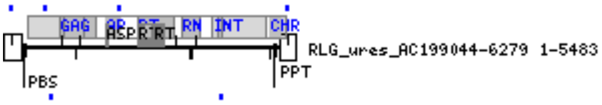

RLC_urum_AC198861-6159

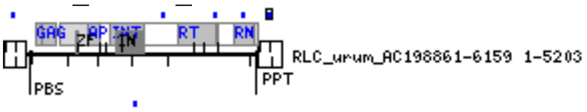

RLG_uvet_AC194145-3904

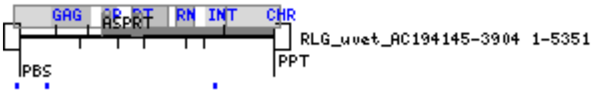

RLG_uwew_AC187787-1947

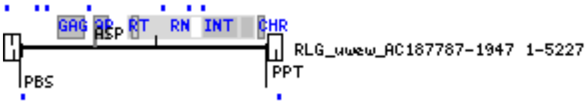

RLC_volo_AC195546-4646

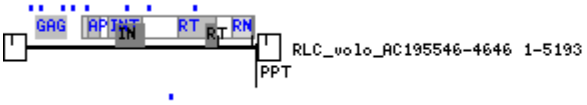

RLC_vuijon_AC194895-4312

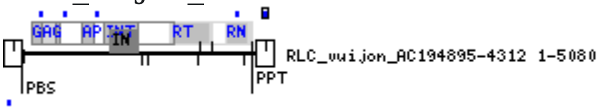

RLX_vuna_AC193505-121

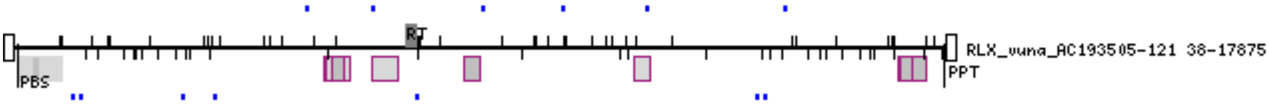

RLC_wamenu_AC191287-3028

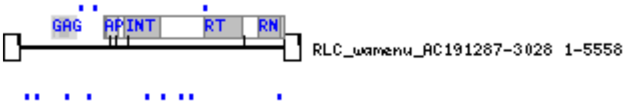

RLG_wemu_AC204680-8396

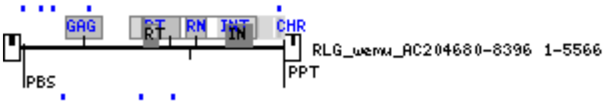

RLC_wiwa_AC191531-3153

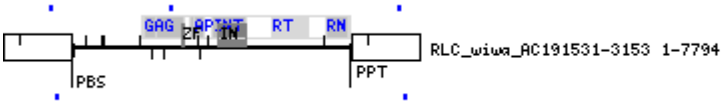

RLC_ydut_AC191122-2965

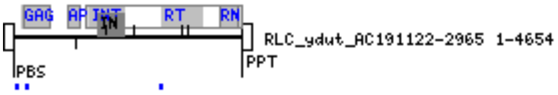

RLC_yrer_AC198936-6221

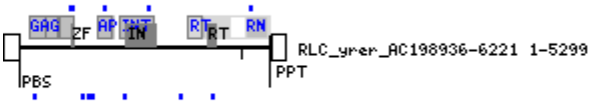

RLC_ytar_AC194428-4096

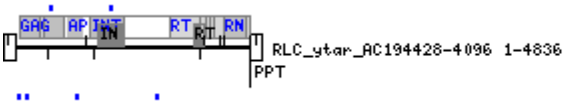

RLG_ytub_AC187411-1880

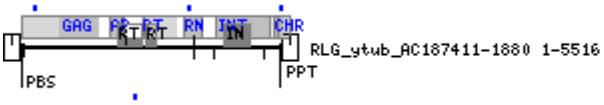

RLG_ywuv_AC197432-5589

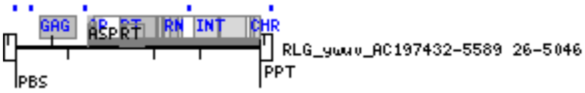

RLC_agep_AC205112-8618

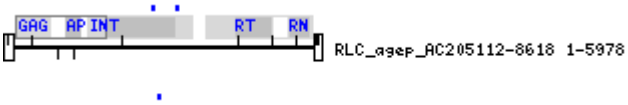

RLG_ahoru_AC187284-1845

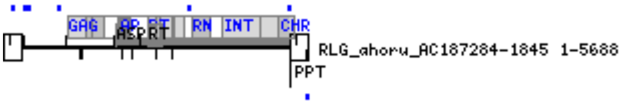

RLC_ajajog_AC191578-3186

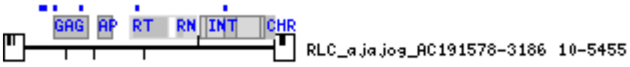

RLG_amiin_AC191769-3319

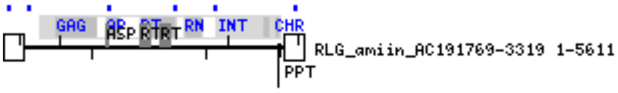

RLG_aneas_AC203312-7773

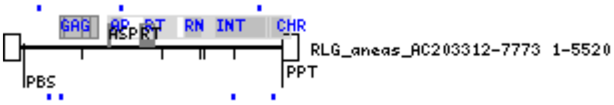

RLG_CRM1_AC191576-3184

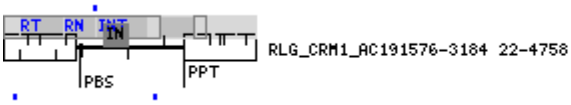

RLG_anysaf_AC203052-7637

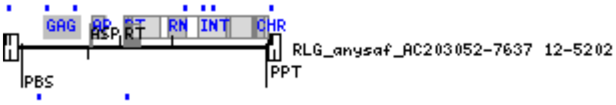

RLG_anysaf_AC211487-11211

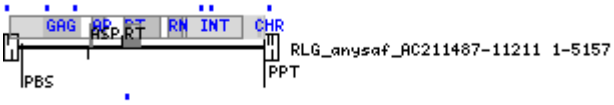

RLG_apil_AC198662-6085

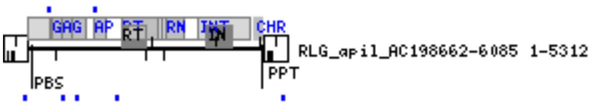

RLG_apil_AC204354-8209

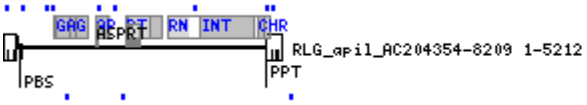

RLG_bobobo_AC201919-7182

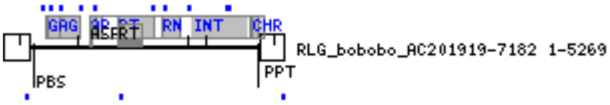

RLG_boha_AC205574-8933

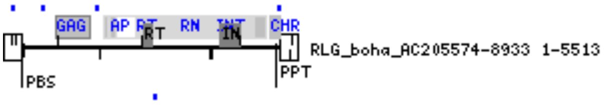

RLG_boja_AC200053-6723

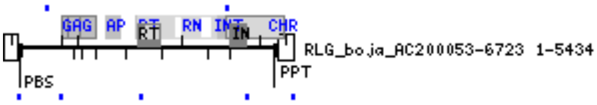

RLG_bosohe_AC185471-1125

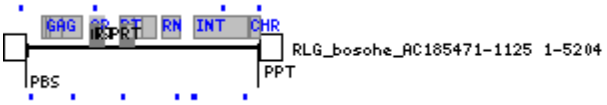

RLG_bosohe_AC190752-2561

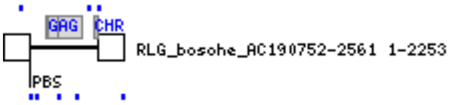

RLG_bosohe_AC191654-3248

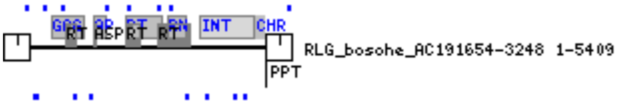

RLG_bosohe_AC205330-8724

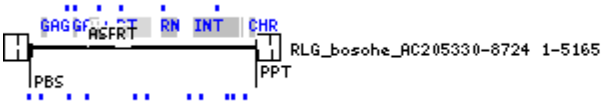

RLG_bosohe_AC205838-9025

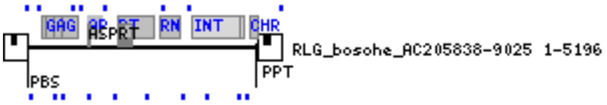

RLG_bosohe_AC206838-9357

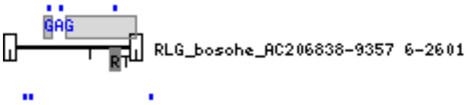

RLG_bosohe_AC212057-11553

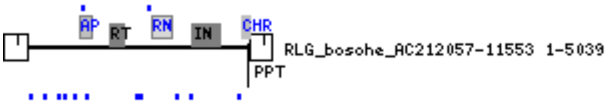

RLG_bosohe_AC213600-12201

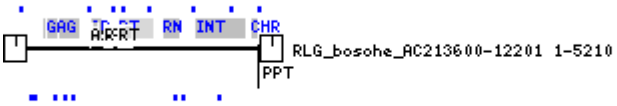

RLG_cinful-zeon_AC177813-189

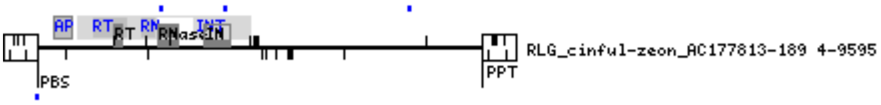

RLG_cinful-zeon_AC177930-407

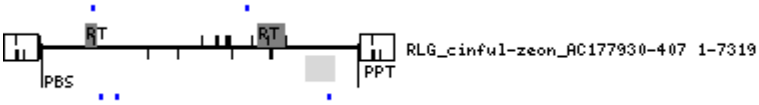

RLG_cinful-zeon_AC183943-734

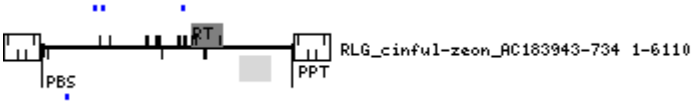

RLG_cinful-zeon_AC186614-1565

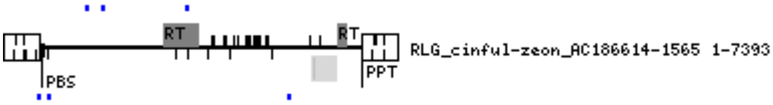

RLG_cinful-zeon_AC191398-3106

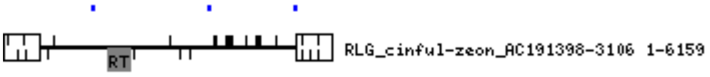

RLG_cinful-zeon_AC191523-3148

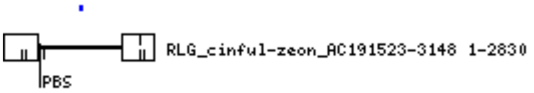

RLG_cinful-zeon_AC192460-3502

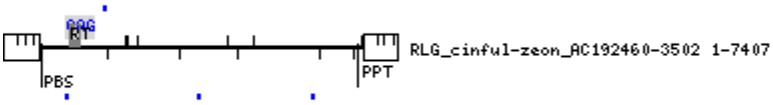

RLG_cinful-zeon_AC193672-3742

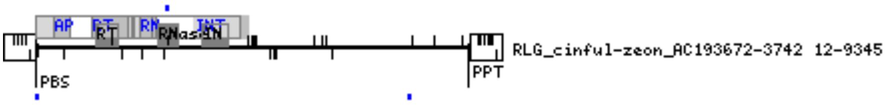

RLG_cinful-zeon_AC194588-4160

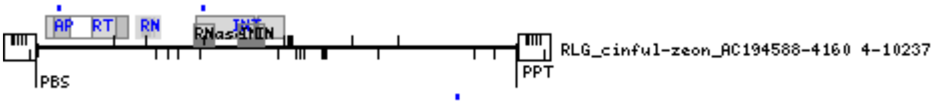

RLG_cinful-zeon_AC194954-4372

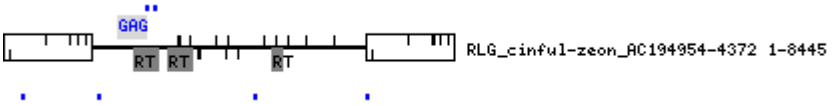

RLG_cinful-zeon_AC194967-4388

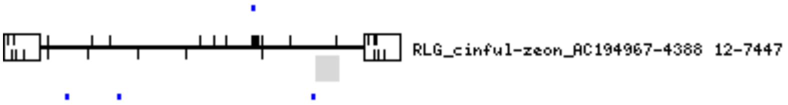

RLG_cinful-zeon_AC195790-4706

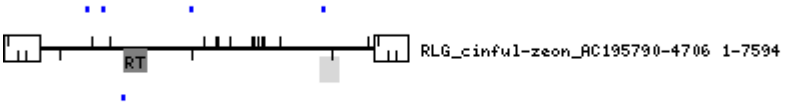

RLG_cinful-zeon_AC198933-6214

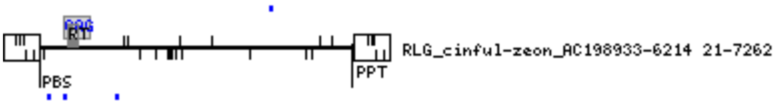

RLG_cinful-zeon_AC199396-6426

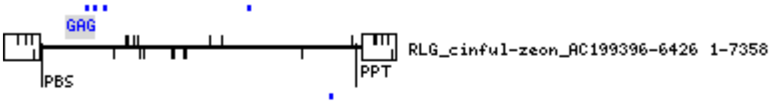

RLG_cinful-zeon_AC199790-6582

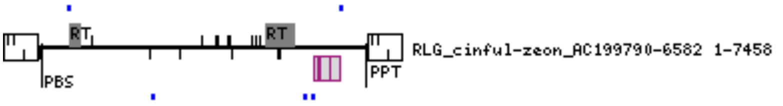

RLG_cinful-zeon_AC199960-6694

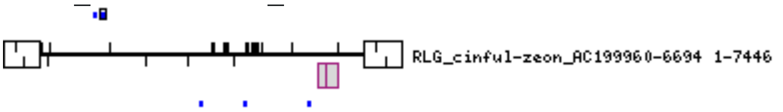

RLG_cinful-zeon_AC200200-6794

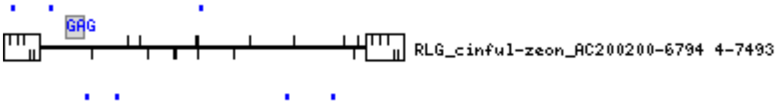

RLG_cinful-zeon_AC202991-7588

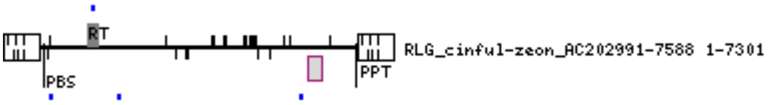

RLG_cinful-zeon_AC203825-7980

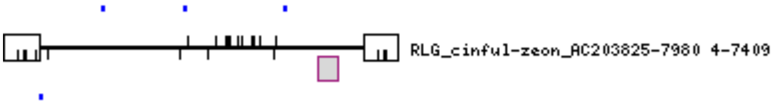

RLG_cinful-zeon_AC205031-8560

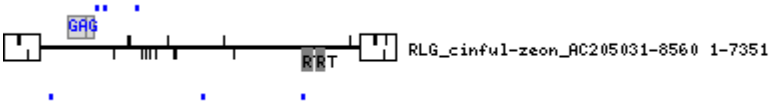

RLG_cinful-zeon_AC205118-8623

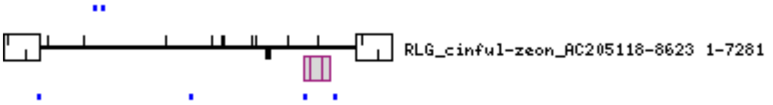

RLG_cinful-zeon_AC206171-9091

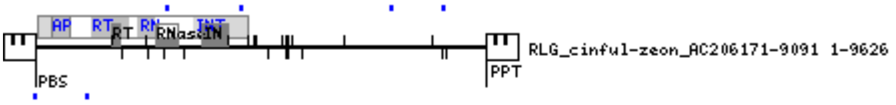

RLG_cinful-zeon_AC206615-9266

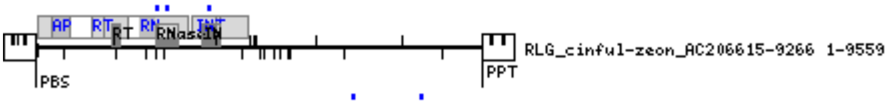

RLG_cinful-zeon_AC207332-9527

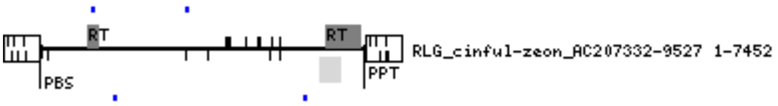

RLG_cinful-zeon_AC207755-9705

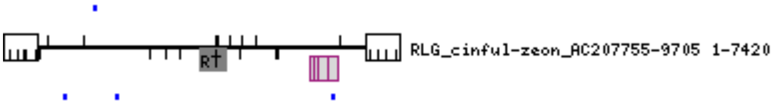

RLG_cinful-zeon_AC208228-9805

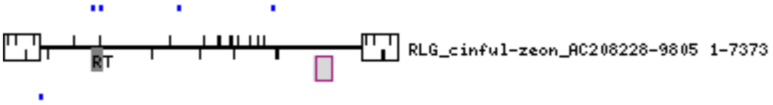

RLG_cinful-zeon_AC208420-9852

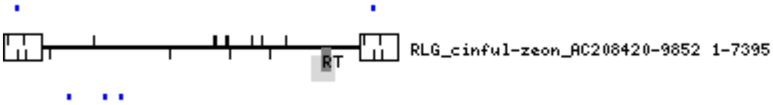

RLG_cinful-zeon_AC208661-9976

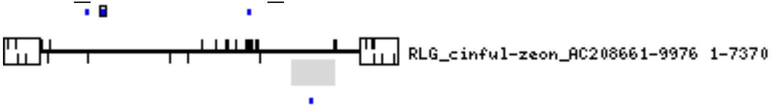

RLG_cinful-zeon_AC209373-10201

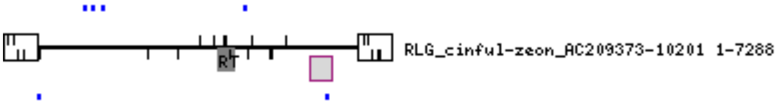

RLG_cinful-zeon_AC211144-11003

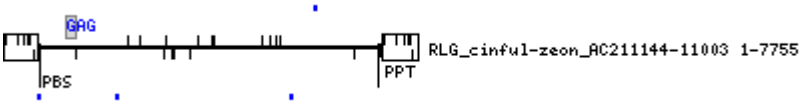

RLG_cinful-zeon_AC211573-11290

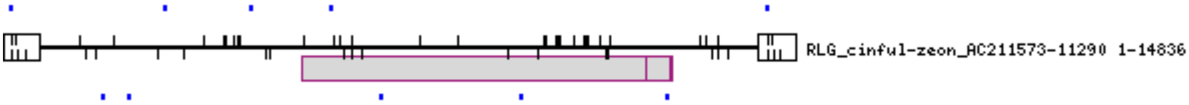

RLG_cinful-zeon_AC212696-11896

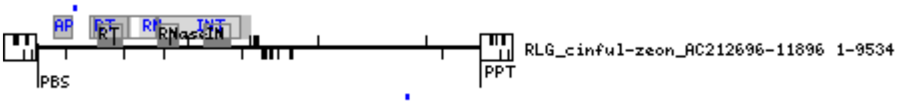

RLG_cinful-zeon_AC213887-12415

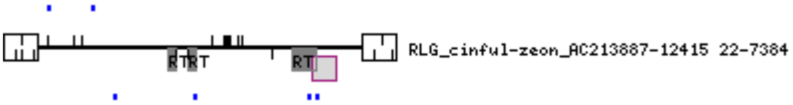

RLG_cinful-zeon_AC215255-13029

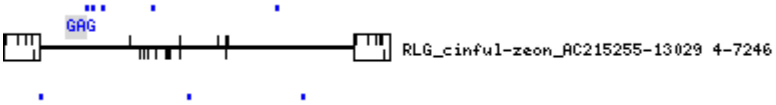

RLG_cinful-zeon_AC216587-13387

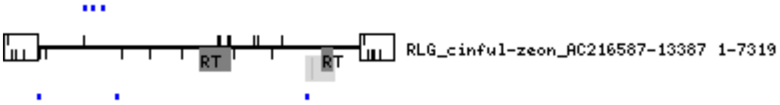

RLG_dagaf_AC182835-556

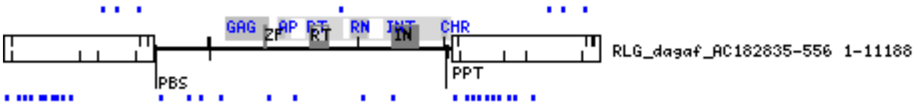

RLG_dagaf_AC195302-4533

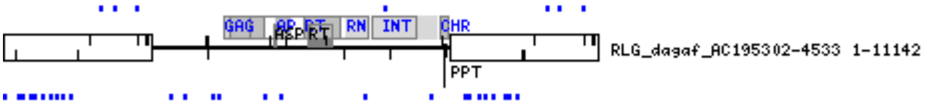

RLG_dagaf_AC208646-9966

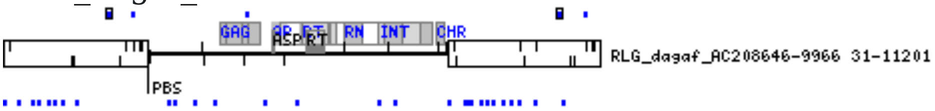

RLC_doke_AC186158-1307

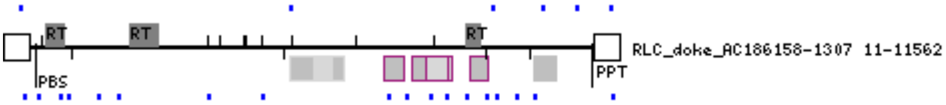

RLG_epohi_AC205903-9050

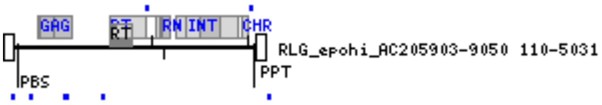

RLG_fege_AC205532-8884

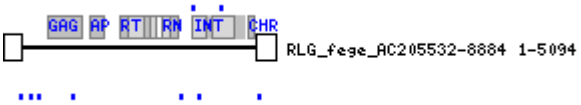

RLC_fourf_AC202975-7570

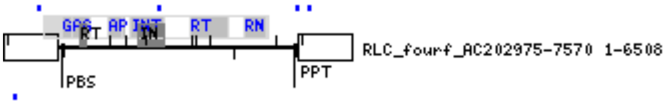

RLG_gati_AC195589-4669

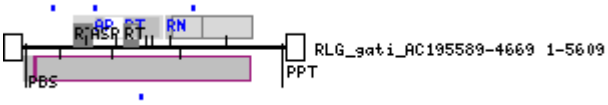

RLC_gilovu_AC196048-4919

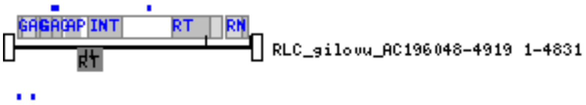

RLG_gofi_AC196116-4977

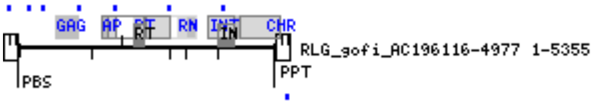

RLG_grande_AC197914-5823

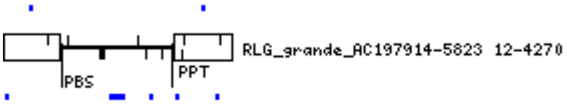

RLG_grande_AC200214-6803

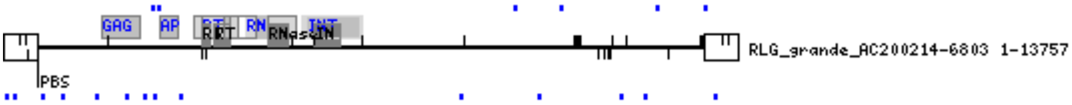

RLG_guhis_AC198413-5999

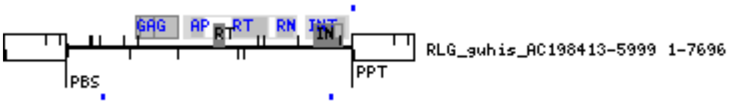

RLG_gyte_AC207411-9568

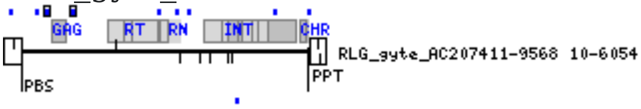

RLC_hera_AC214536-12775

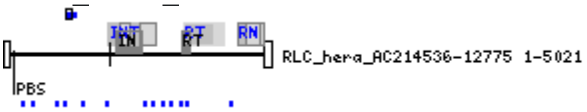

RLC_homy_AC197914-5822

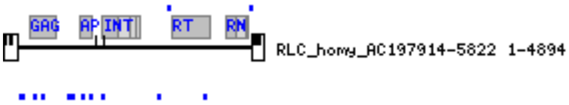

RLG_huck_AC186603-1556

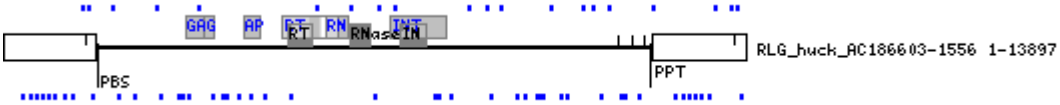

RLG_huck_AC186656-1609

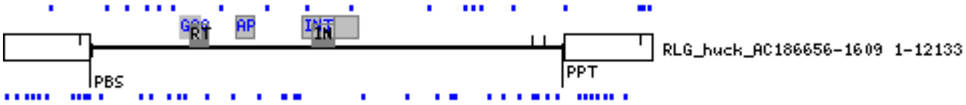

RLG_huck_AC190900-2713

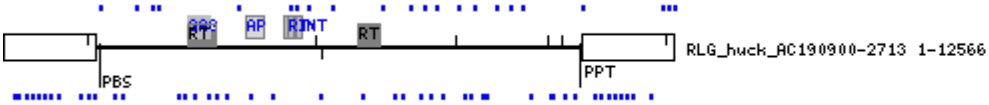

RLG_huck_AC191259-3001

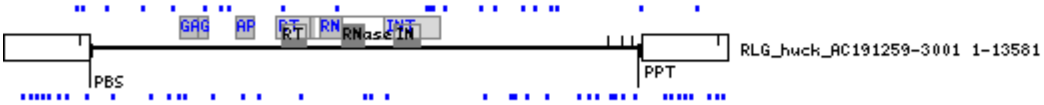

RLG_huck_AC193313-3542

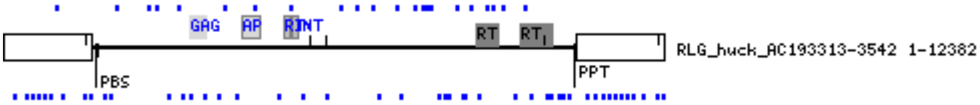

RLG_huck_AC194973-4393

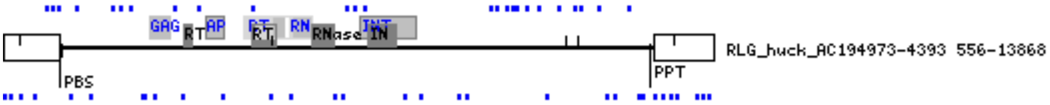

RLG_huck_AC195575-4652

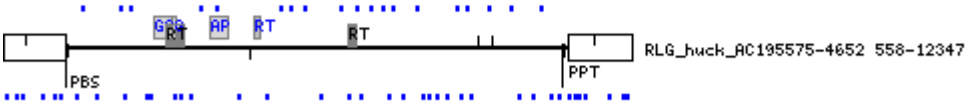

RLG_huck_AC199444-6460

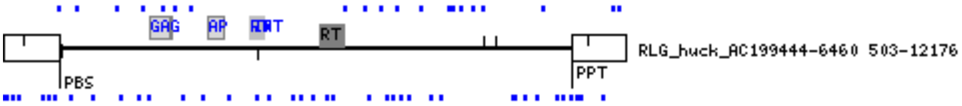

RLG_huck_AC208546-9913

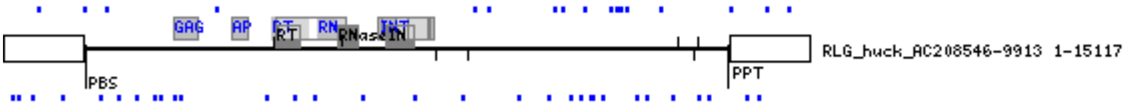

RLG_huck_AC208842-10038

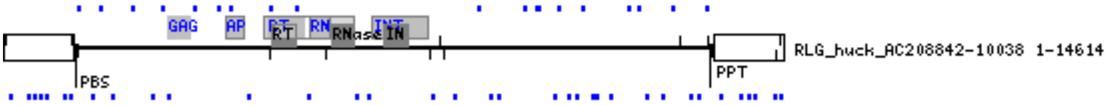

RLG_huck_AC210079-10574

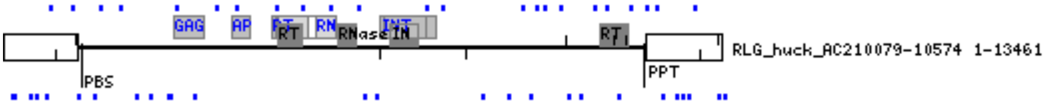

RLG_huck_AC210804-10865

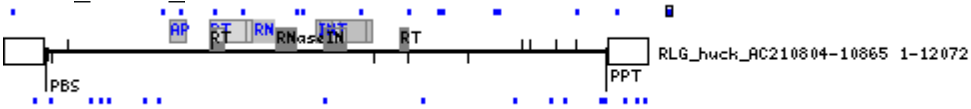

RLG_huck_AC213612-12218

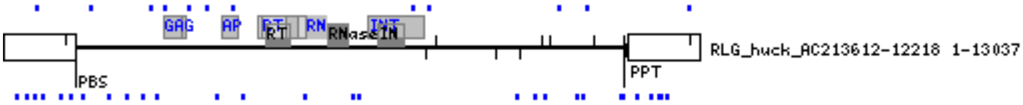

RLG_huck_AC214833-12913

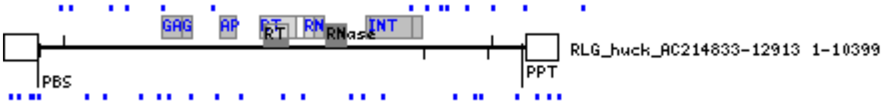

RLG_huck_AC216048-13250

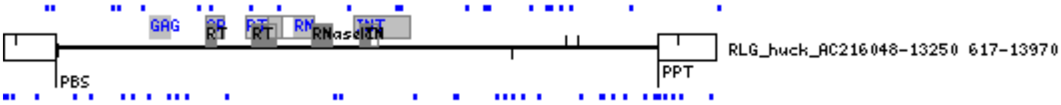

RLC_iseb_AC207352-9544

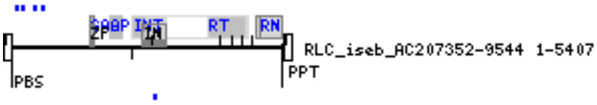

RLG_ivuk_AC194103-3869

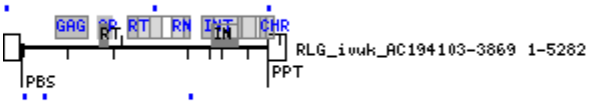

RLC_janoov_AC209690-10294

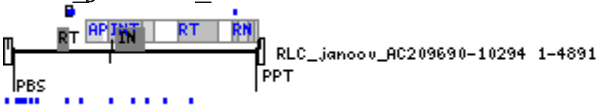

RLG_jaws_AC186187-1320

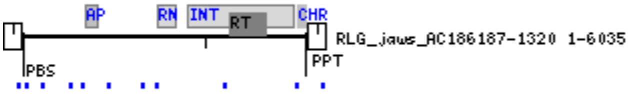

RLG_jaws_AC192382-3471

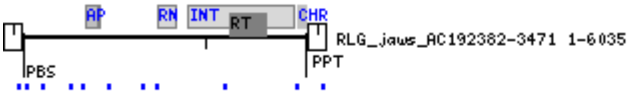

RLG_kubi_AC195480-4624

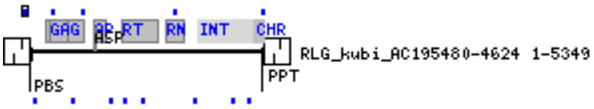

RLG_kubi_AC196180-5016

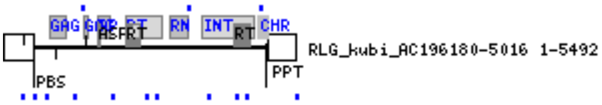

RLG_laiwa_AC214288-12602

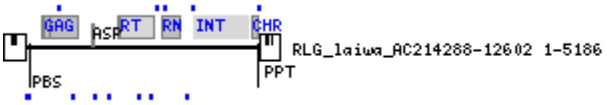

RLG_lata_AC191117-2960

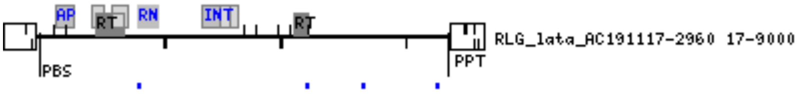

RLG_lyruom_AC185669-1267

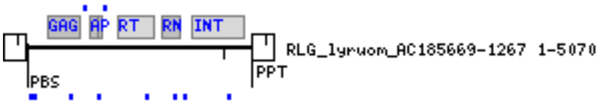

RLG_moorud_AC199961-6697

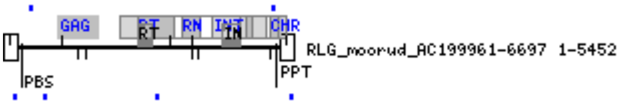

RLG_mufeub_AC194206-3950

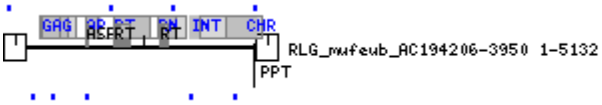

RLC_naasuj_AC211569-11281

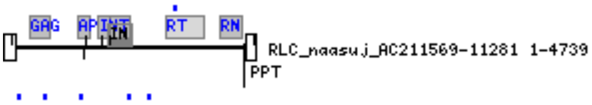

RLG_naijaj_AC205556-8907

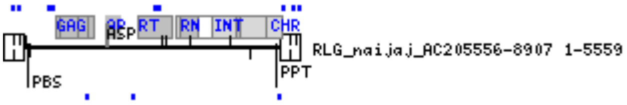

RLG_nakovu_AC206324-9191

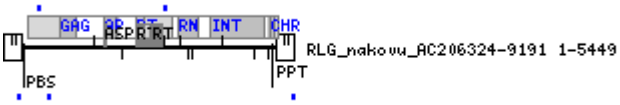

RLC_neha_AC215285-13046

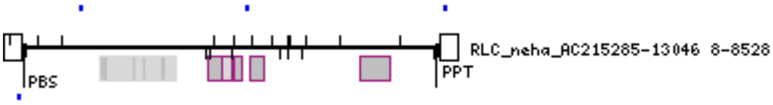

RLG_nobe_AC198224-5924

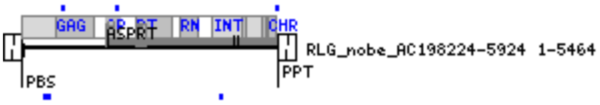

RLG_nopip_AC188036-2052

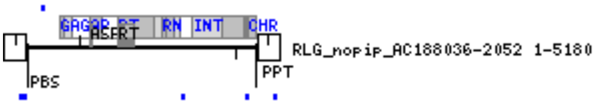

RLG_ojam_AC198400-5989

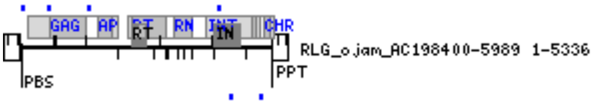

RLG_ojav_AC202435-7427

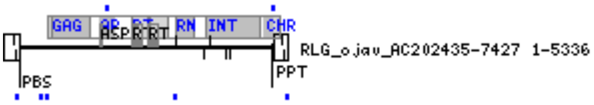

RLG_okopam_AC187789-1948

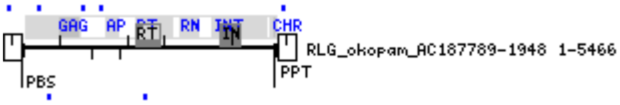

RLC_omud_AC203533-7881

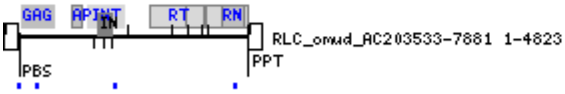

RLC_onal_AC186648-1594

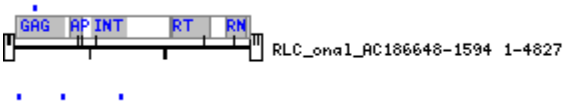

RLG_oveah_AC206282-9165

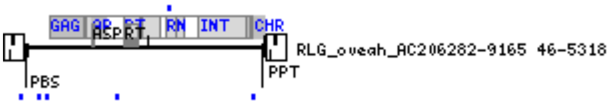

RLG_pebi_AC207888-9755

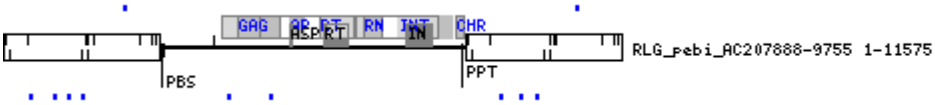

RLG_piube_AC183505-603

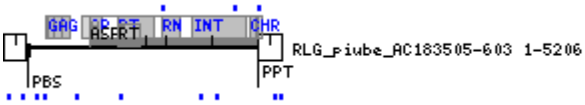

RLG_prem1_AC186287-1362

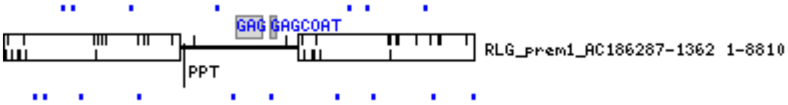

RLG_prem1_AC196065-4927

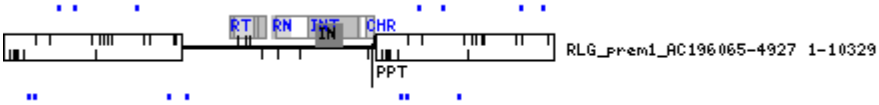

RLG_prem1_AC200105-6751

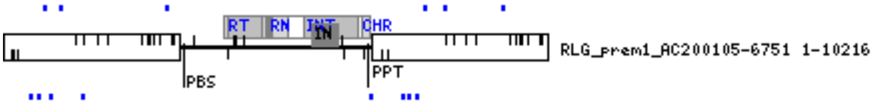

RLG_prem1_AC201801-7095

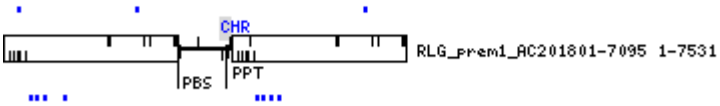

RLG_prem1_AC206253-9147

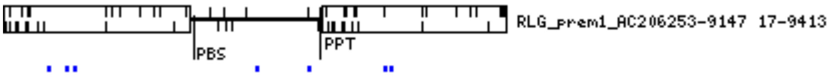

RLG_prem1_AC212325-11702

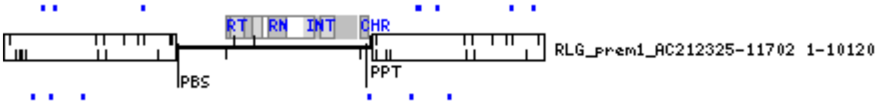

RLG_prem1_AC215184-12949

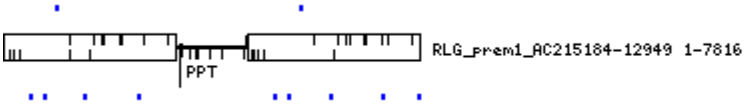

RLX_rijuep_AC188965-2150

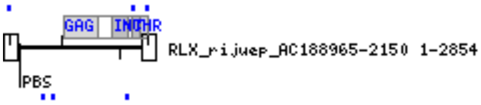

RLG_rimaar_AC197236-5493

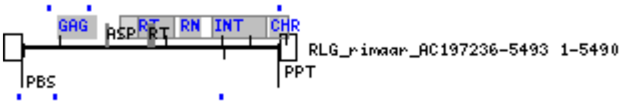

RLG_rowi_AC203429-7862

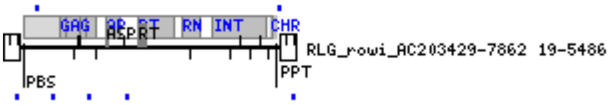

RLG_ruugu_AC214105-12477

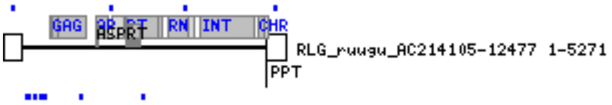

RLG_satulo_AC197006-5311

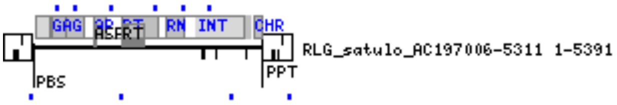

RLC_sehoad_AC198380-5972

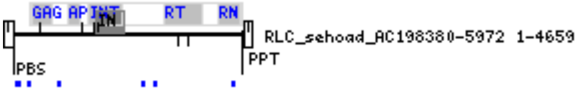

RLC_seko_AC188710-2086

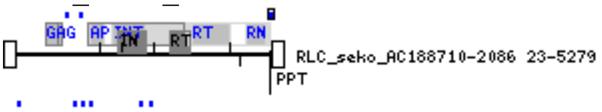

RLG_sokiit_AC210743-10848

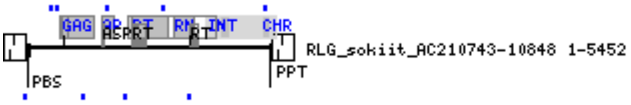

RLG_suda_AC215200-12975

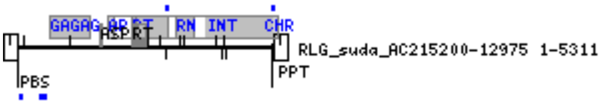

RLG_sywu_AC183899-694

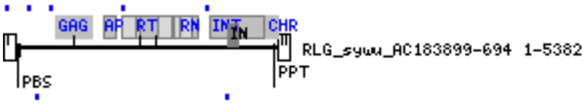

RLG_taro_AC203167-7686

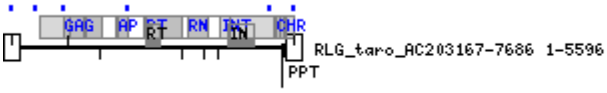

RLG_tituer_AC196493-5158

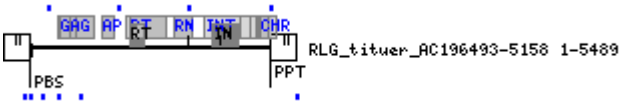

RLG_ubid_AC210086-10583

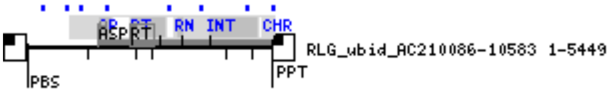

RLG_udokup_AC210420-10735

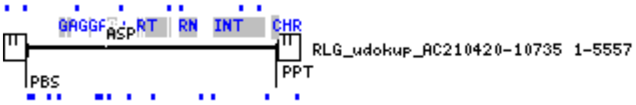

RLG_umojev_AC204420-8262

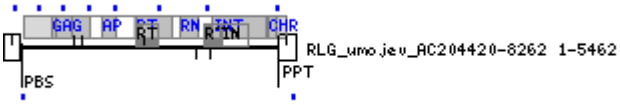

RLG_usif_AC206781-9344

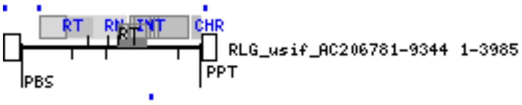

RLG_usuf_AC208533-9903

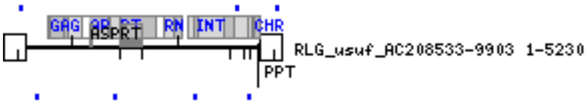

RLC_uwaf_AC197569-5671

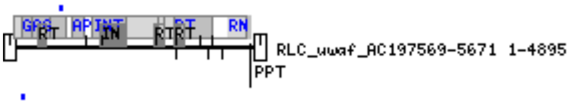

RLC_vodida_AC203384-7831

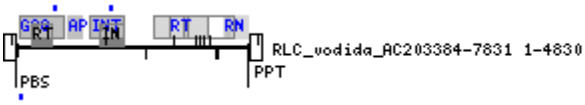

RLG_vufi_AC198989-6257

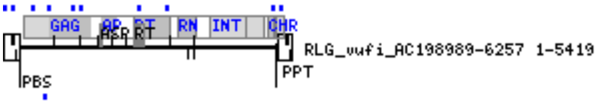

RLG_waepo_AC190953-2782

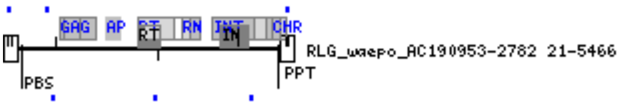

RLG_weaniv_AC196414-5097

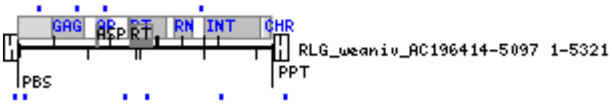

RLG_weki_AC194431-4099

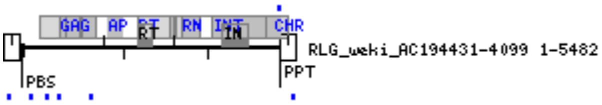

RLG_yfages_AC197085-5383

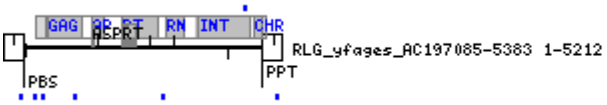

RLG_ywyt_AC209975-10517

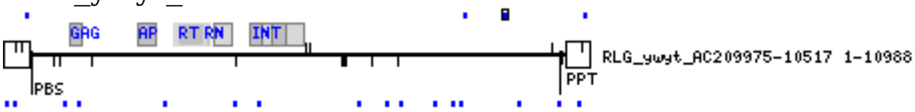

RLG_abiri_AC203571-7891

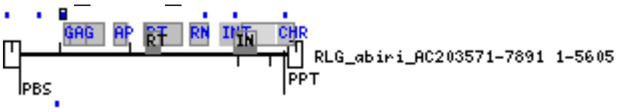

RLX_adun_AC193506-122

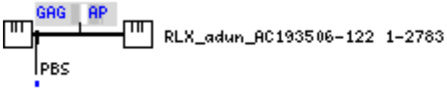

RLC_ahov_AC193643-3726

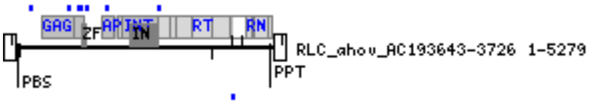

RLX_ajeb_AC188141-66

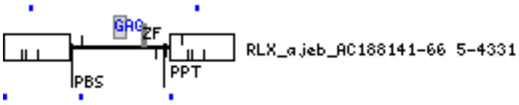

RLC_atej_AC187217-40

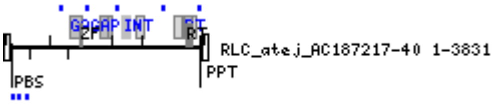

RLX_avahi_AC191363-3084

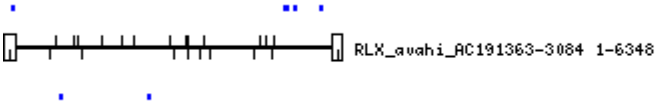

RLG_awuhe_AC190897-2710

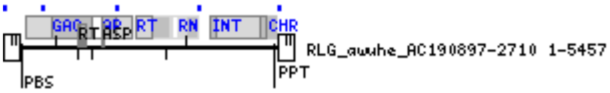

RLX_baha_AC187214-39

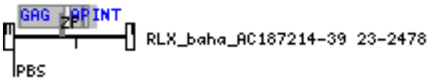

RLX_bawigu_AC208532-9902

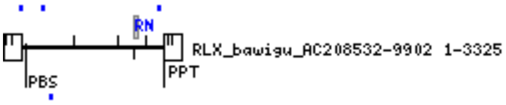

RLX_beboso_AC190260-76

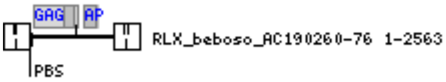

RLX_beby_AC214433-12667

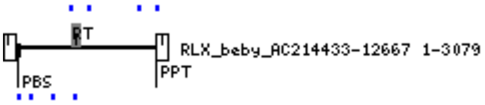

RLX_bene_AC198379-5969

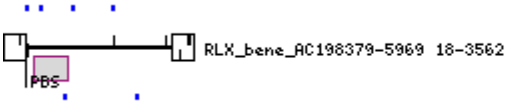

RLX_beva_AC190814-90

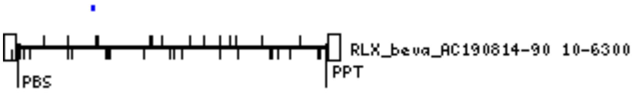

RLX_biwa_AC194319-131

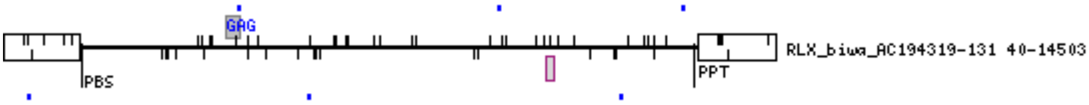

RLG_bobeg_AC193485-3670

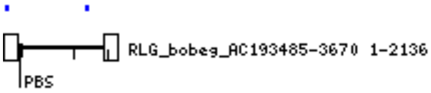

RLG_bogu_AC196148-4995

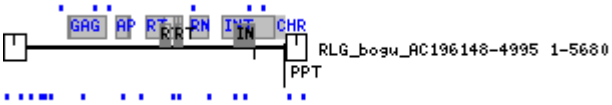

RLG_bogu_AC198870-6180

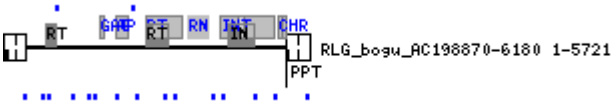

RLX_bori_AC190960-100

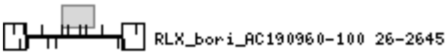

RLX_bosovu_AC193480-119

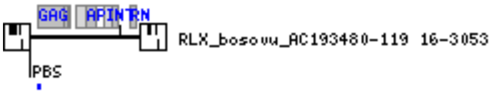

RLX_bs1_AC185605-1189

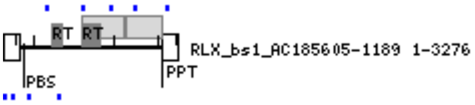

RLX_bs1_AC208724-10016

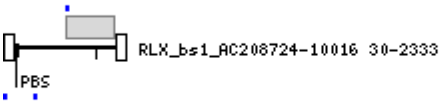

RLX_bumy_AC216354-13379

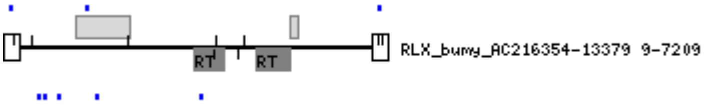

RLX_CRM2_AC206920-9397

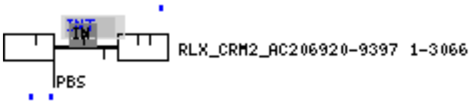

RLG_dabe_AC212399-11783

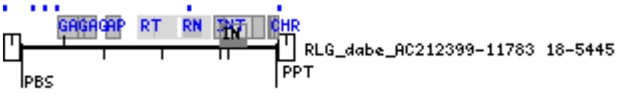

RLX_dady_AC210684-10809

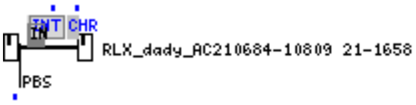

RLX_dala_AC216254-13336

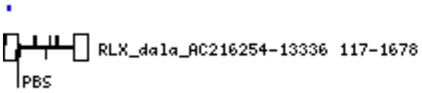

RLX_defub_AC191393-106

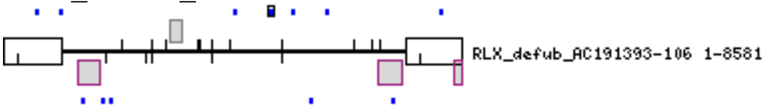

RLX_demo_AC202036-173

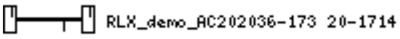

RLX_doba_AC190263-77

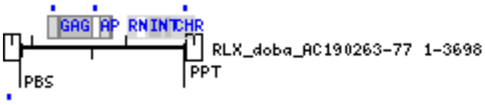

RLC_ebel_AC210216-10670

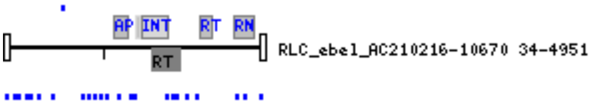

RLC_ebel_AC211737-11397

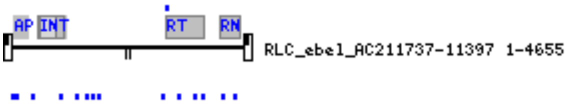

RLC_ebel_AC213044-12072

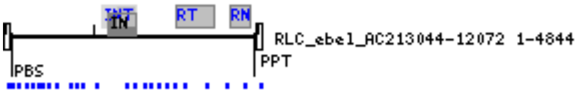

RLX_efaw_AC190815-91

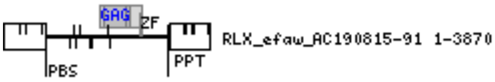

RLX_emuh_AC193462-157

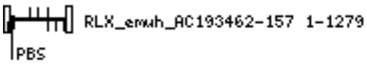

RLX_epiil_AC190716-83

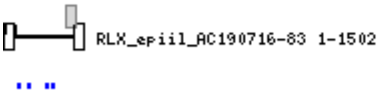

RLX_etin_AC194319-129

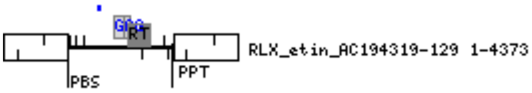

RLX_etug_AC187099-1770

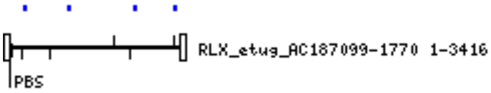

RLX_CRM3_AC200048-6717

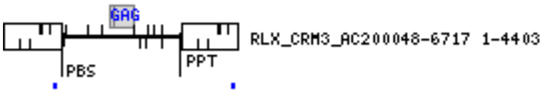

RLG_ewib_AC198384-5975

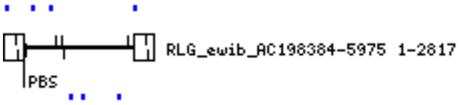

RLG_ewib_AC207533-9599

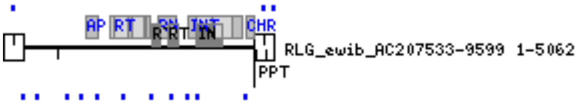

RLX_ewigyw_AC185312-18

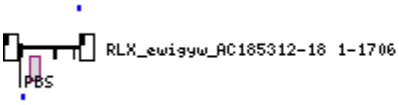

RLG_ewog_AC212715-11905

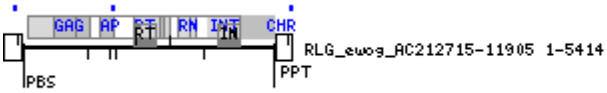

RLX_ewot_AC210077-10573

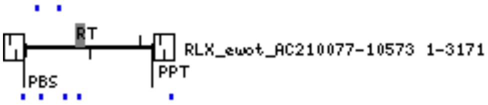

RLX_ewuof_AC188199-68

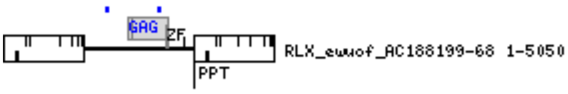

RLX_fajy_AC190874-2682

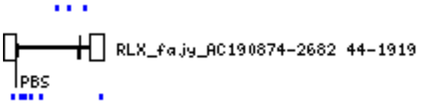

RLX_fanuab_AC193594-3712

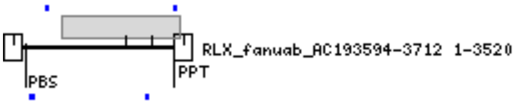

RLX_fara_AC188142-67

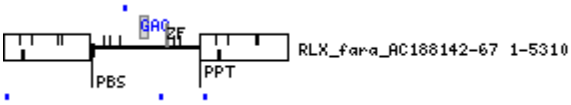

RLX_fate_AC194466-4144

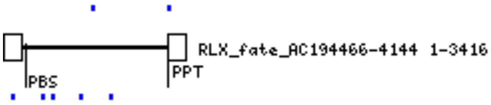

RLC_fehod_AC184774-851

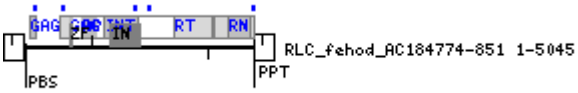

RLC_fuvej_AC194215-3955

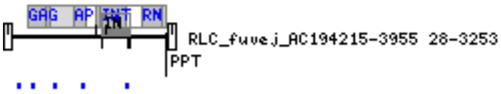

RLX_gate_AC197186-143

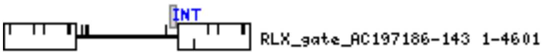

RLC_gekog_AC203828-7984

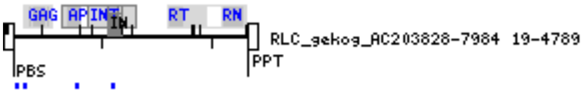

RLX_geta_AC187066-33

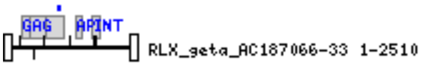

RLC_giream_AC204000-8116

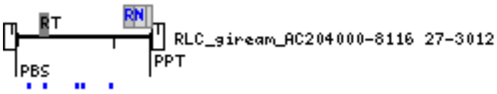

RLX_gotur_AC201794-7086

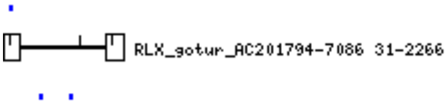

RLX_guafa_AC188016-60

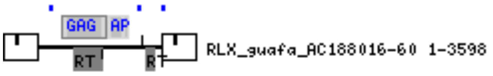

RLX_guali_AC190263-78

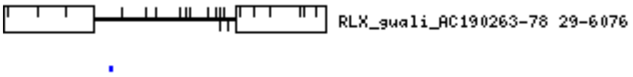

RLX_gufa_AC194066-3854

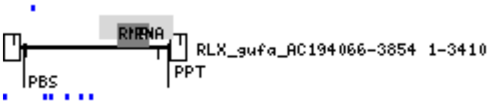

RLC_gugu_AC193480-120

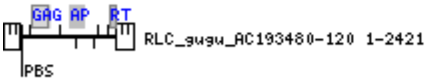

RLC_guwo_AC183661-638

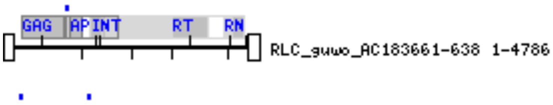

RLX_halo_AC205330-8725

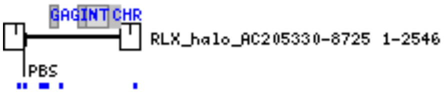

RLC_hani_AC186285-1359

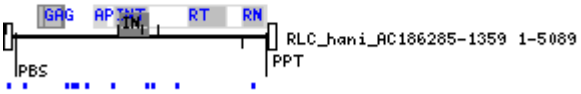

RLC_hesa_AC204349-8207

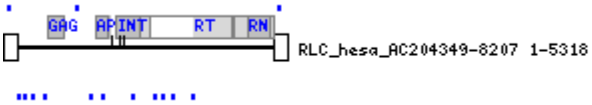

RLC_hiri_AC187471-44

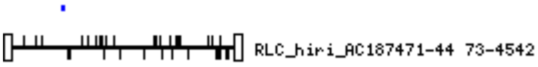

RLX_hoda_AC193645-3730

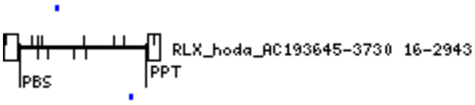

RLG_hooni_AC211474-11202

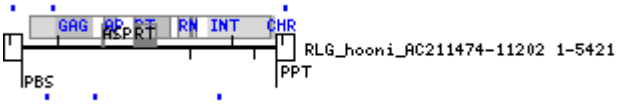

RLC_hopscotch_AC209396-10229

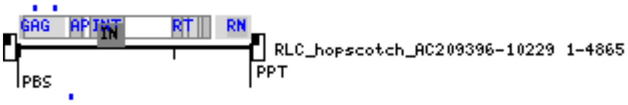

RLC_huti_AC197153-5449

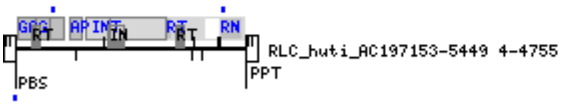

RLX_hutu_AC210780-10851

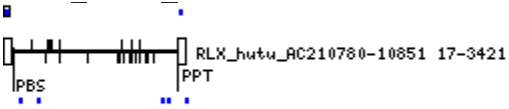

RLX_ifab_AC209906-10473

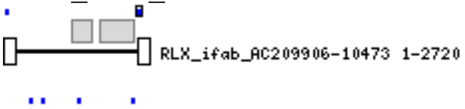

RLX_ilofaw_AC186598-1545

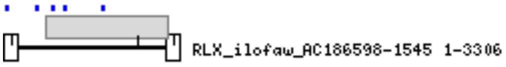

RLC_japov_AC213985-12447

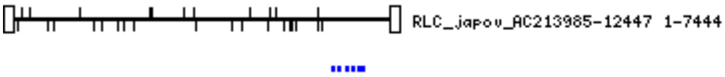

RLX_jeli_AC200611-6933

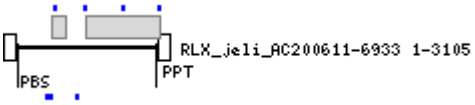

RLX_jeli_AC208705-10001

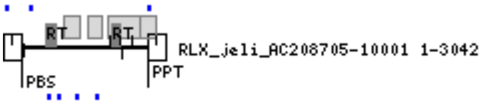

RLX_joemon_AC177891-147

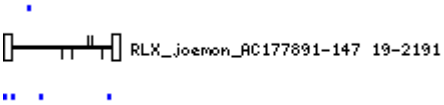

RLX_jupek_AC208518-9894

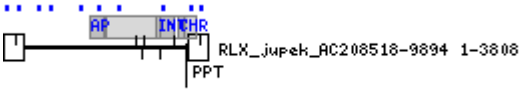

RLX_juta_AC190498-2318

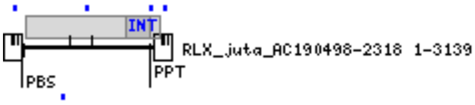

RLC_kake_AC186604-1559

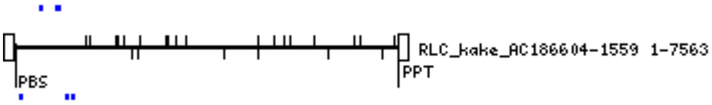

RLX_kawivo_AC186793-30

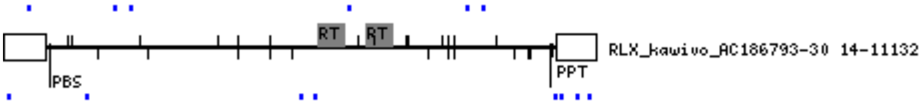

RLX_kinosi_AC183924-714

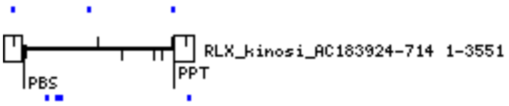

RLC_lafa_AC205893-9045

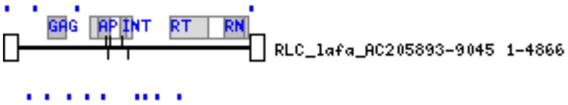

RLX_lamyab_AC208713-10008

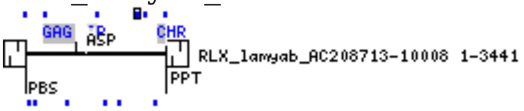

RLX_leso_AC215500-13080

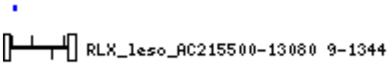

RLG_loba_AC194942-4364

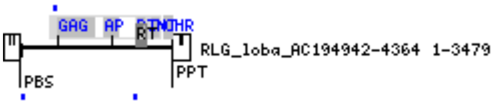

RLX_loukuv_AC197842-5799

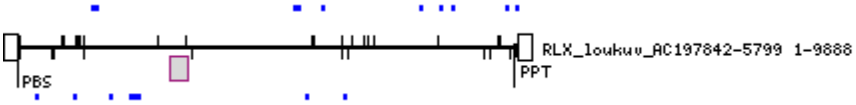

RLX_lyna_AC194093-3868

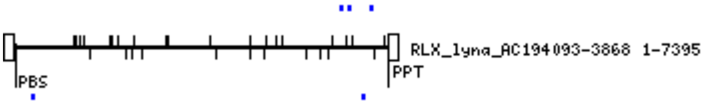

RLG_puck_AC208456-9876

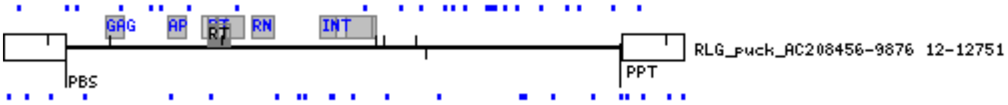

RLG_puck_AC208673-9982

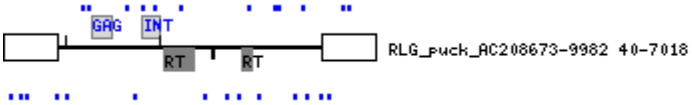

RLG_puck_AC211927-11478

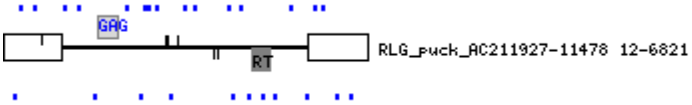

RLG_puck_AC215312-13067

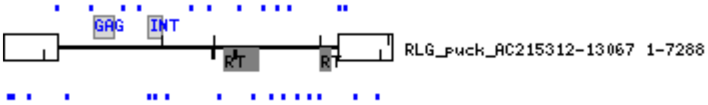

RLX_mafigi_AC216705-13396

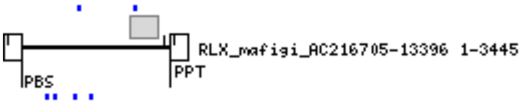

RLX_mafogo_AC199961-6695

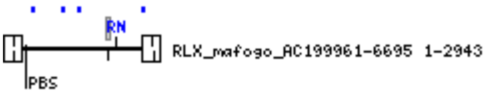

RLG_magellan_AC194897-4313

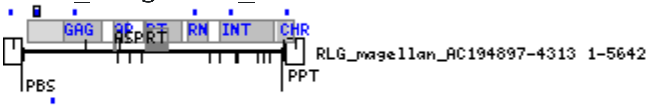

RLX_mako_AC200748-6991

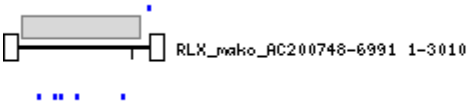

RLX_maono_AC208340-9832

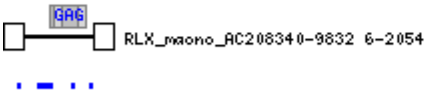

RLG_maro_AC204359-8211

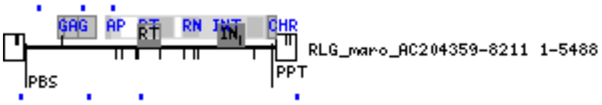

RLX_mibaab_AC205139-8652

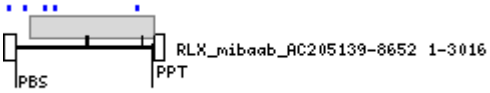

RLX_milt_AC194936-4356

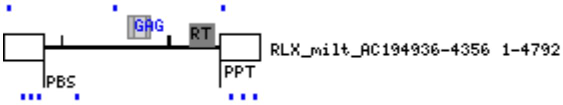

RLX_milt_AC198975-6250

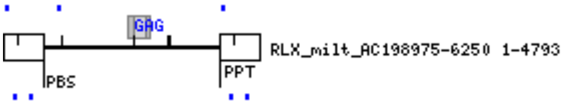

RLX_milt_AC209648-10275

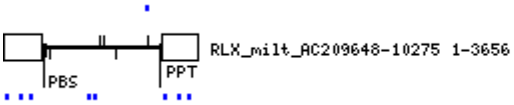

RLX_miva_AC188030-62

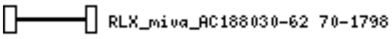

RLX_mopin_AC187657-153

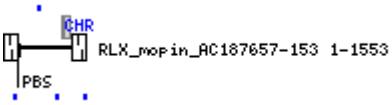

RLX_mopin_AC194135-3897

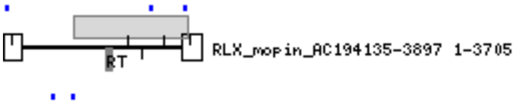

RLX_mopin_AC197230-5485

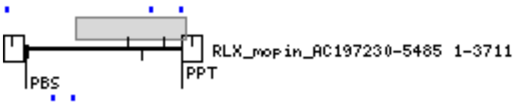

RLC_muekeh_AC184787-871

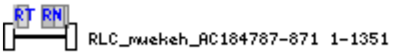

RLX_mulaf_AC207733-9686

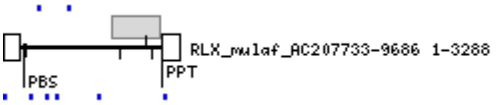

RLX_muusi_AC190834-92

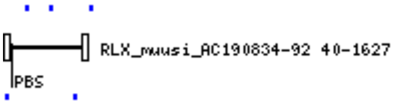

RLG_CRM4_AC201761-7053

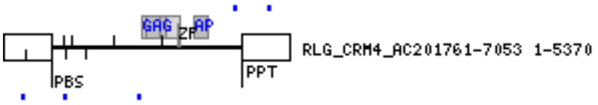

RLG_CRM4_AC206864-9376

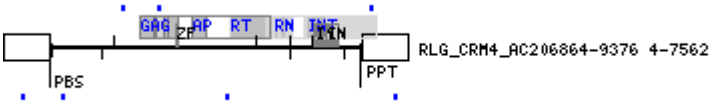

RLX_nabu_AC187471-45

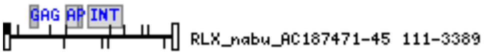

RLX_nabu_AC187882-57

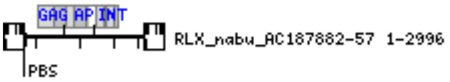

RLX_naiba_AC195481-139

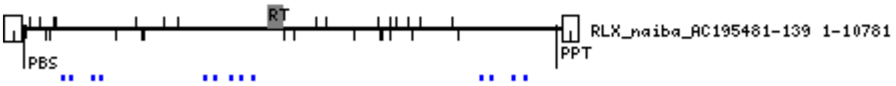

RLX_nakuuv_AC199045-169

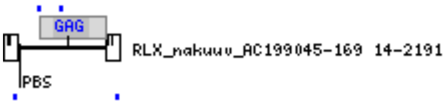

RLX_name_AC197689-5725

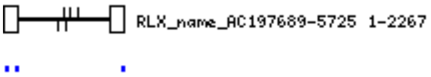

RLX_nana_AC209373-10202

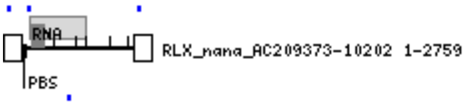

RLG_nasi_AC205509-8857

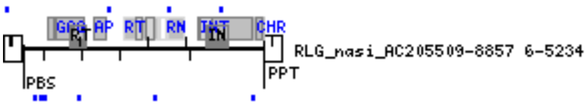

RLX_neafu_AC185666-1261

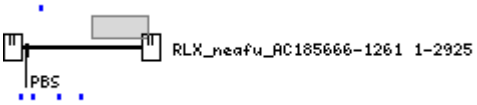

RLX_neteut_AC188982-2172

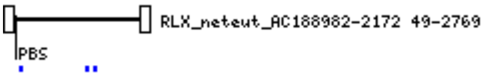

RLX_nisow_AC208616-9947

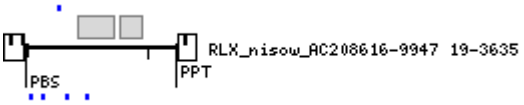

RLC_nitat_AC190618-2442

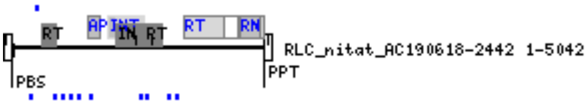

RLX_niypo_AC191087-2935

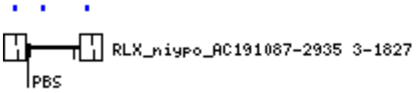

RLC_nuhan_AC206272-9161

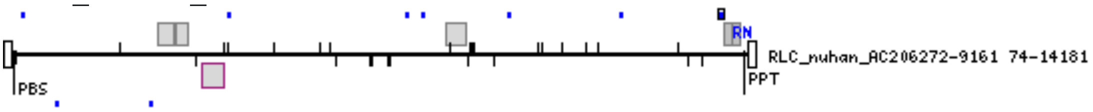

RLX_nyjuvy_AC200867-7004

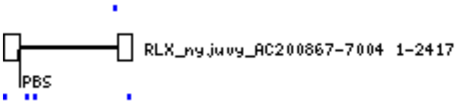

RLX_nyjuvy_AC212394-11768

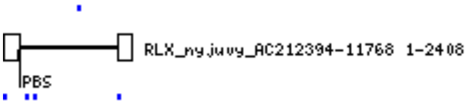

RLX_odip_AC216266-13348

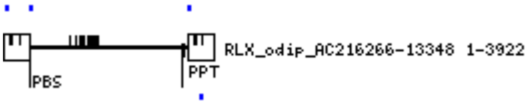

RLC_ogiv_AC205856-9034

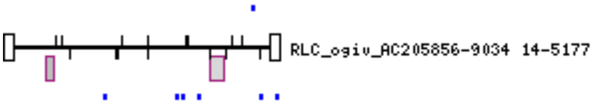

RLX_ohag_AC187801-48

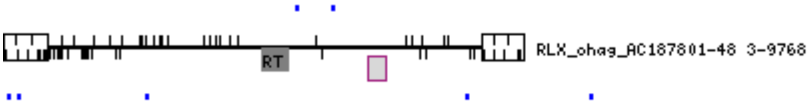

RLX_ojah_AC184792-875

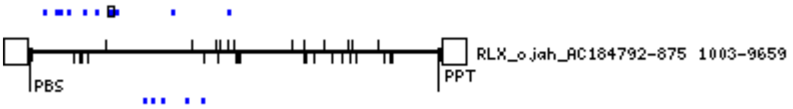

RLX_okoj_AC198787-167

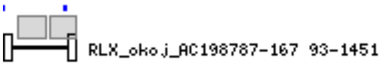

RLX_okur_AC194642-135

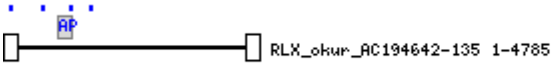

RLG_olepo_AC212207-11645

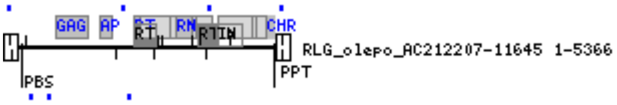

RLX_onub_AC190523-2356

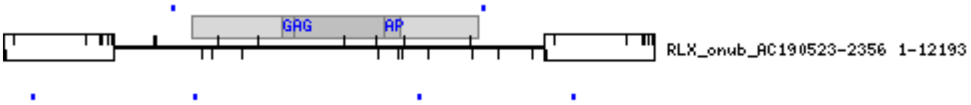

RLX_osed_AC191084-2931

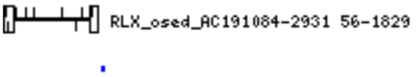

RLG_ovamef_AC196977-5281

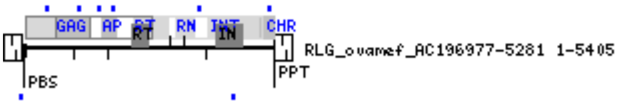

RLX_ovev_AC199878-6641

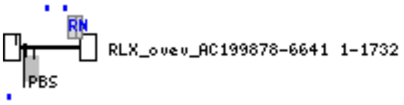

RLX_oviil_AC187836-55

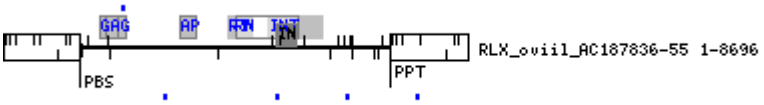

RLX_ovys_AC187066-34

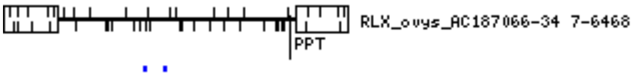

RLX_oweiw_AC190860-2662

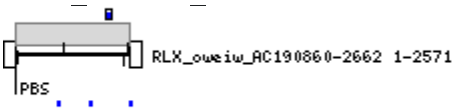

RLG_pagof_AC198204-5908

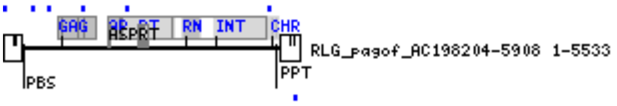

RLX_panen_AC192606-115

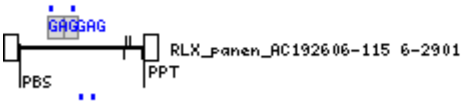

RLX_petopi_AC195376-4582

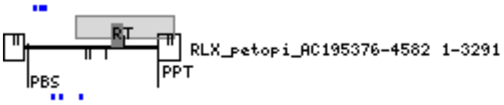

RLX_pibo_AC201915-172

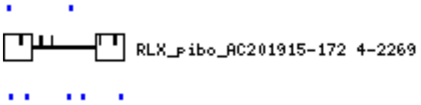

RLX_poarow_AC200533-6899

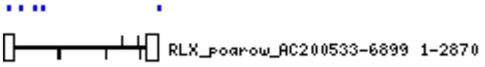

RLX_pope_AC205335-180

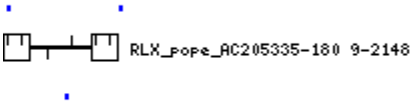

RLC_pute_AC197188-5467

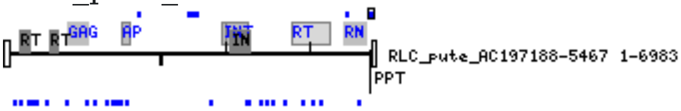

RLX_raga_AC196386-5066

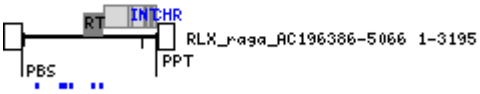

RLG_reina_AC200147-6773

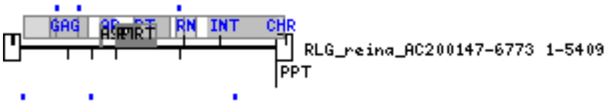

RLX_rulo_AC186371-28

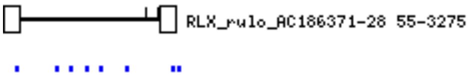

RLX_ruwi_AC204382-8227

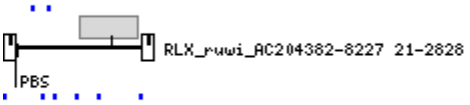

RLX_saahol_AC198506-6044

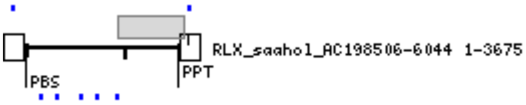

RLX_sari_AC184117-11

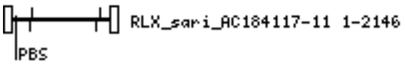

RLX_sego_AC188397-69

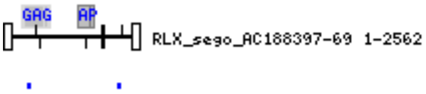

RLX_sela_AC195130-4415

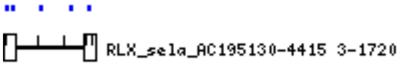

RLX_sido_AC204367-8219

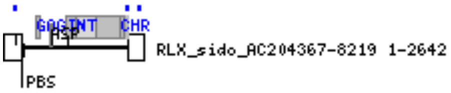

RLX_soefes_AC212121-11599

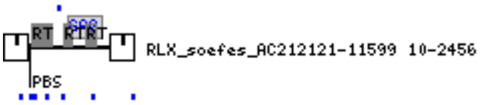

RLC_sofi_AC215261-13035

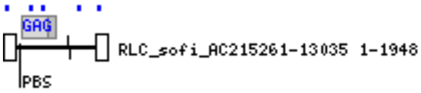

RLG_tekay_AC211245-11065

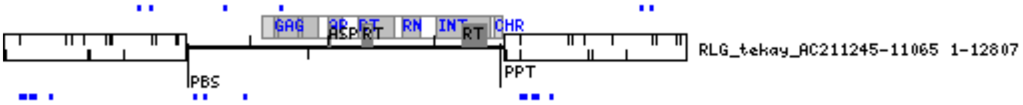

RLX_teki_AC202867-7492

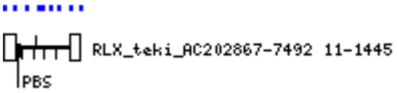

RLX_tojena_AC187554-1910

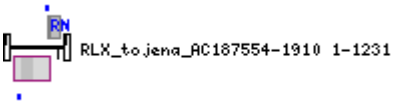

RLX_toro_AC197199-5471

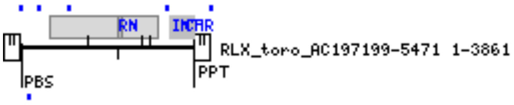

RLG_tuku_AC177929-405

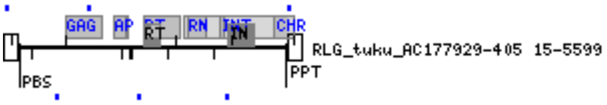

RLX_tuteh_AC183372-584

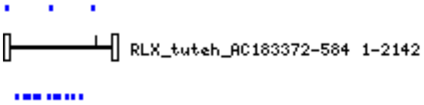

RLX_tywo_AC187810-53

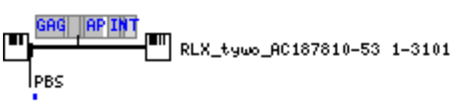

RLC_ubel_AC183941-731

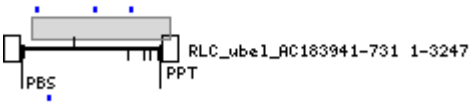

RLC_ubel_AC194376-4060

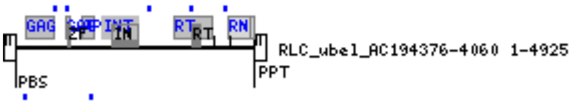

RLX_ubow_AC194933-4355

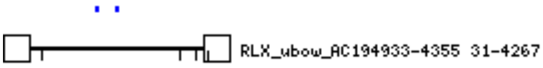

RLG_ugano_AC216043-13241

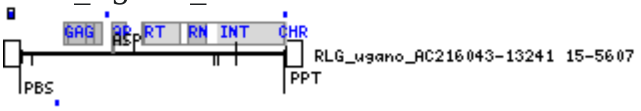

RLX_ujinas_AC217279-13469

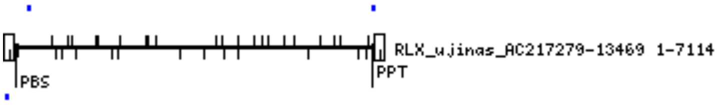

RLX_ukov_AC204842-177

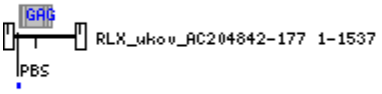

RLC_uloh_AC209080-10120

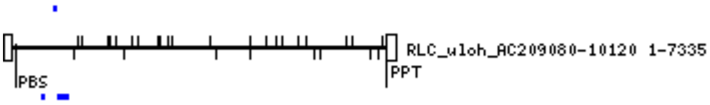

RLX_ulon_AC190608-155

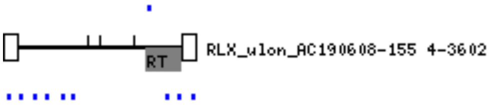

RLG_uper_AC185487-1151

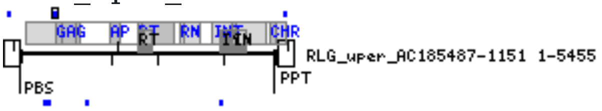

RLG_upus_AC200875-7012

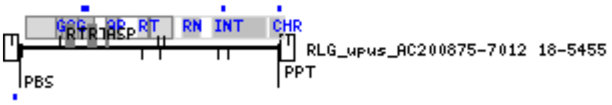

RLX_utar_AC212228-11664

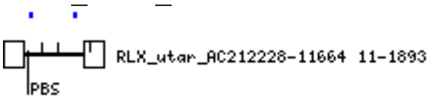

RLX_uvis_AC197719-164

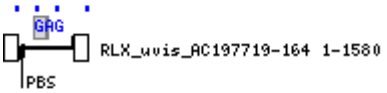

RLX_uwub_AC195372-161

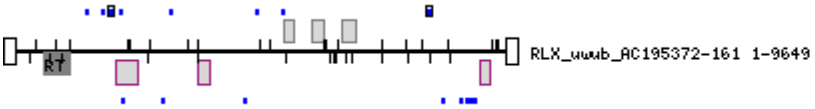

RLG_uwum_AC177933-415

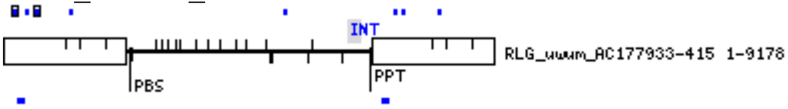

RLG_uwum_AC190887-2701

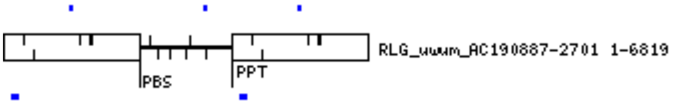

RLG_uwum_AC213069-12092

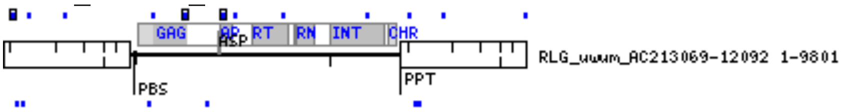

RLG_uwuw_AC198589-6052

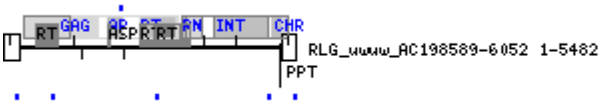

RLX_vafim_AC196676-5176

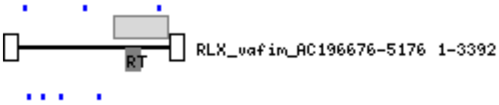

RLX_vaofen_AC202996-7597

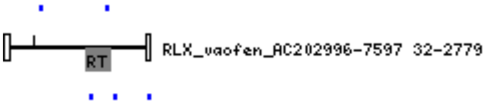

RLX_vedi_AC198992-6258

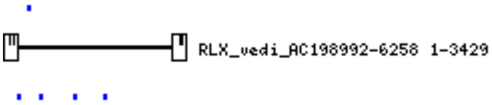

RLX_vegu_AC190718-85

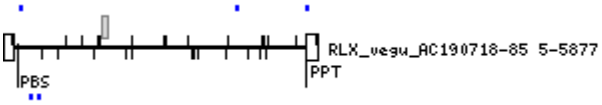

RLX_vora_AC206187-9112

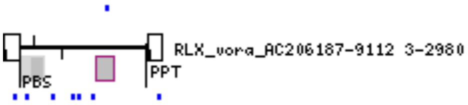

RLX_vufe_AC194263-159

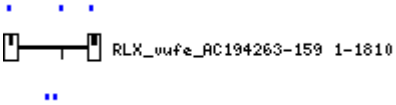

RLX_vusu_AC187081-1751

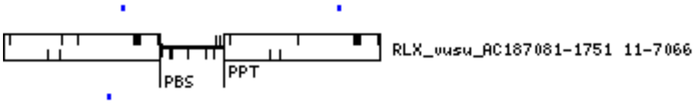

RLX_wawu_AC211017-10990

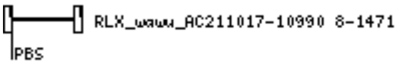

RLX_wihov_AC205351-8739

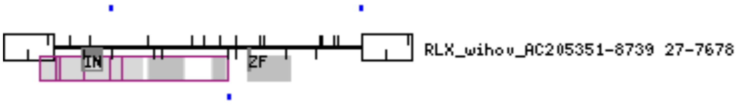

RLX_wiolus_AC210058-10567

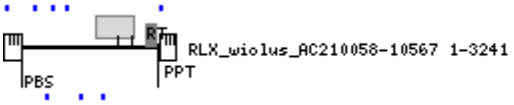

RLX_wiru_AC210670-10789

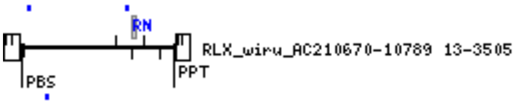

RLG_witi_AC184140-796

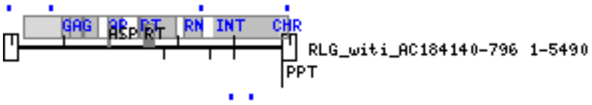

RLX_wugaab_AC187472-46

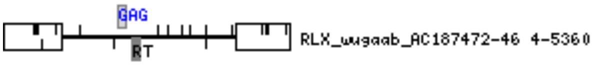

RLG_wuwe_AC212122-11600

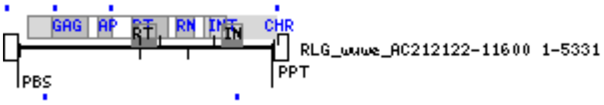

RLG_wyly_AC198779-6150

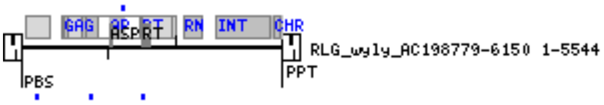

RLX_yemi_AC201773-7064

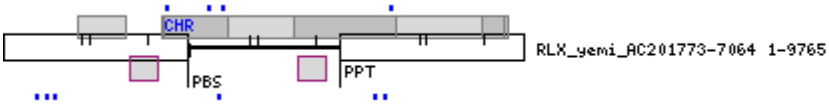

RLX_yraj_AC205486-8834

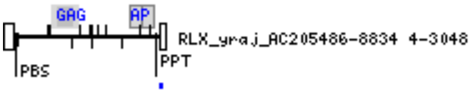

RLX_yreud_AC198385-5976

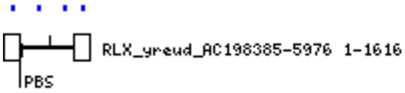

RLG_yvoj_AC215638-13124

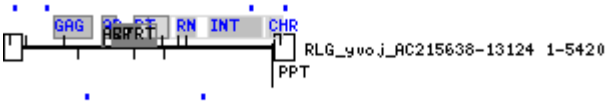

RLX_ywely_AC190897-98

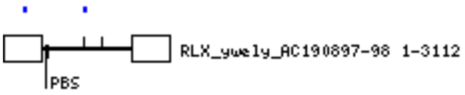

RLC_votaed_AC215881-13209

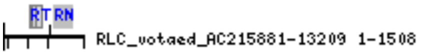

RLG_cinful-zeon_AC194347-4039

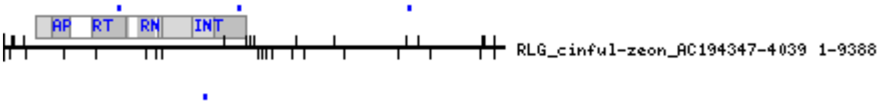

RLG_cinful-zeon_AC203004-7602

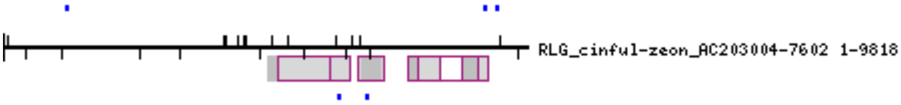

RLG_cinful-zeon_AC205768-9013

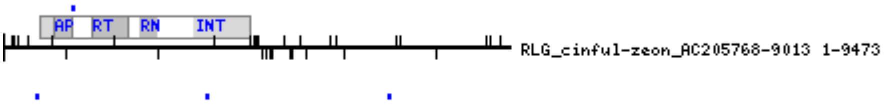

RLG_cinful-zeon_AC210140-10614

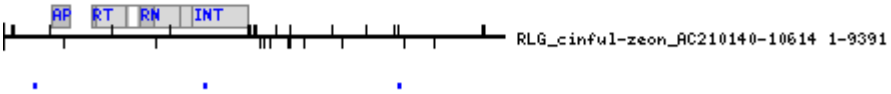

RLG_CRM1_AC207803-9728

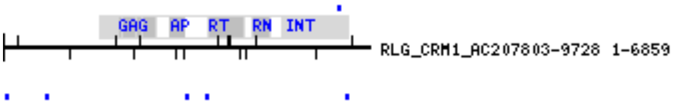

RLG_CRM1_AC208678-9984

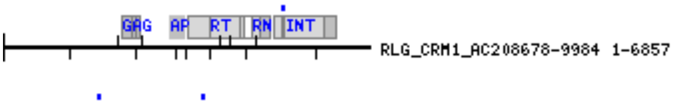

RLG_ewiut_AC194106-3871

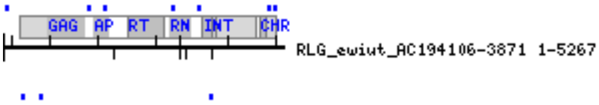

RLG_huck_AC199418-6452

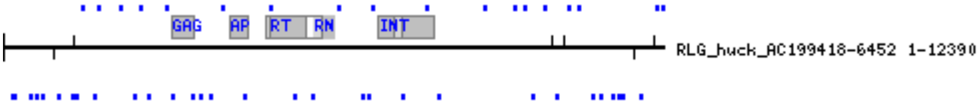

RLG_huck_AC203007-7610

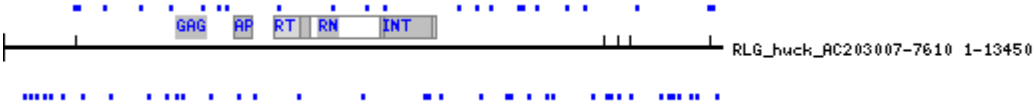

RLG_huck_AC212331-11708

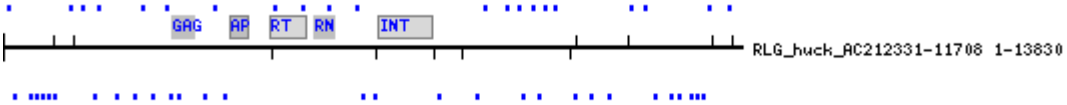

RLG_huck_AC213042-12069

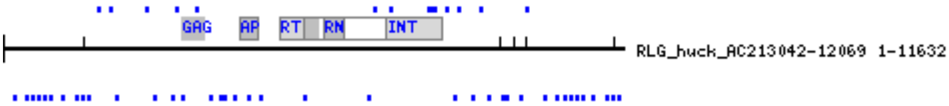

RLG_prem1_AC184142-802

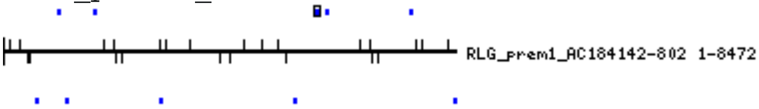

RLG_prem1_AC191715-3282

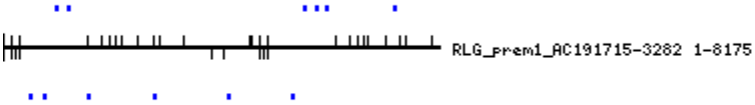

RLG_prem1_AC200740-6986

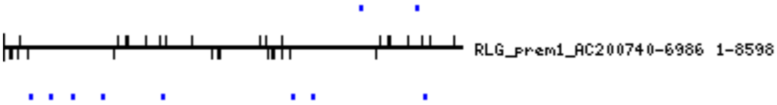

RLG_xilon-diguus_AC195486-4629

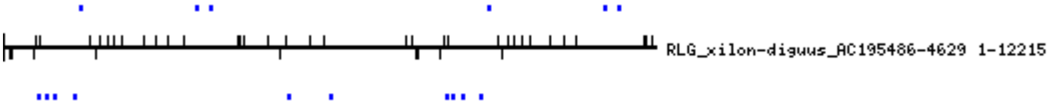

RLG_xilon-diguus_AC203313-7774

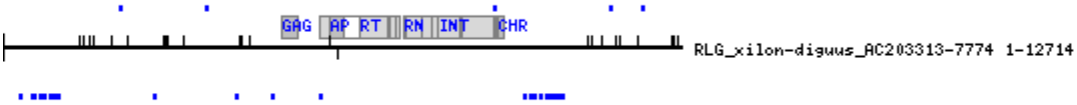

RLX_jube_AC191704-110

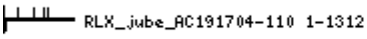

RLX_lenu_AC213984-12443

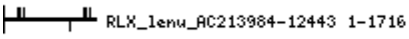

RLX_milt_AC211742-11402

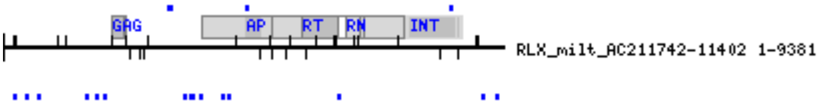

Supplement: Supplementary file 1 — Annotation of all 579 maize TEs included in this study. The presence and position of detectable LTRs, PBS and PPT sequences (LTR Finder), protein-coding domains (BLASTX) and potential quadruplex sequences (PQS; pqsfinder). White rectangles represent LTRs, blue rectangles are common TE domains (labelled) or other domains detected in Uniprot (unlabelled). Small blue bars are PQS with score > 24 (> 64 larger bar). (PDF 927 kb) [file 12864_2018_4563_MOESM1_ESM.pdf]
