# Supplementary figures and images for: Quadruplex DNA in long terminal repeats in maize LTR retrotransposons inhibits the expression of a reporter gene in yeast
Source: BMC Genomics. 2018 Mar 6;19:184. doi: 10.1186/s12864-018-4563-7 (PMC5838962; doi:10.1186/s12864-018-4563-7)

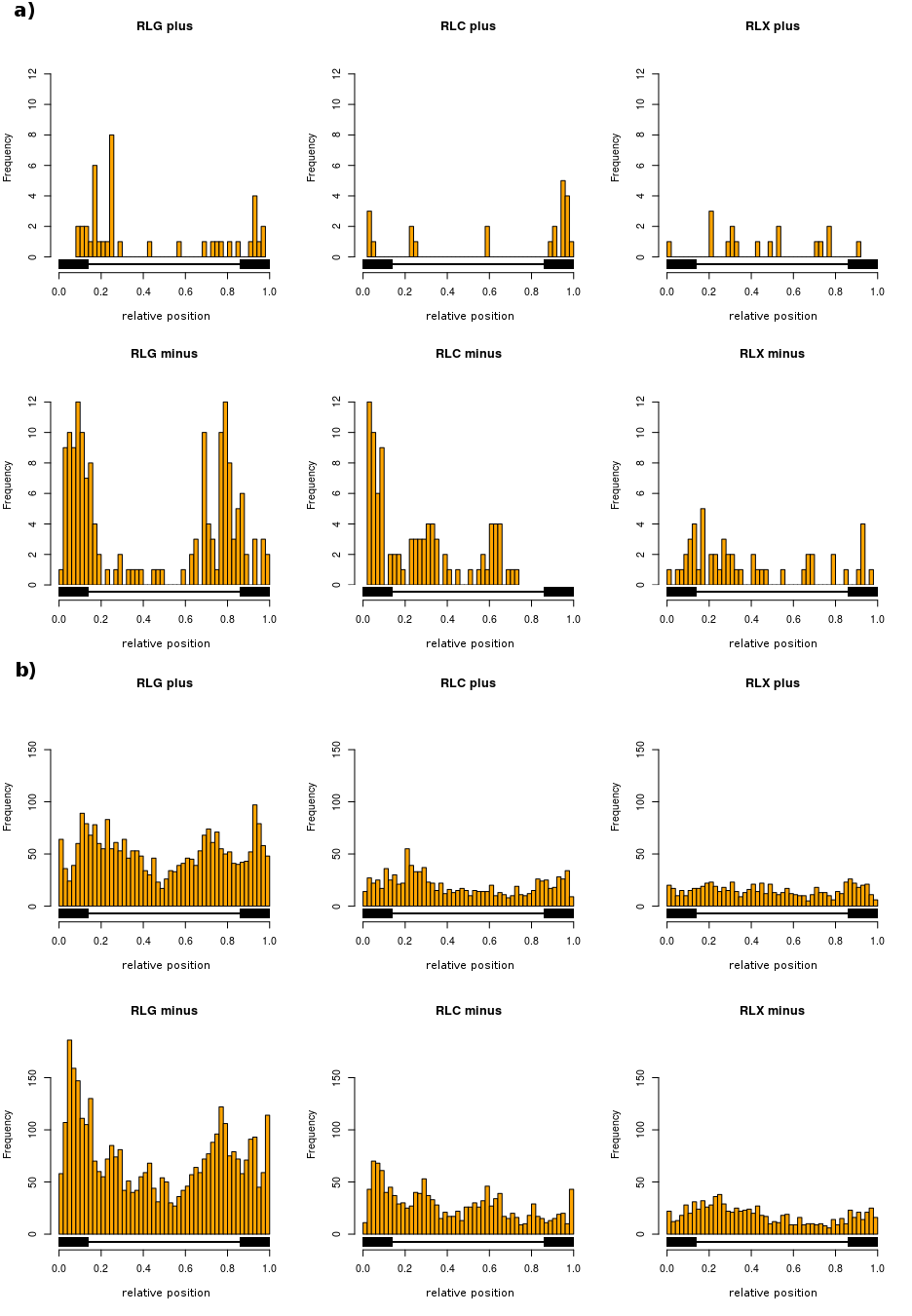

Supplement: Supplementary file 3 — Occurrence of high-scoring PQS along maize LTR retrotransposons. The distribution of PQS containing a minimum of four adequately spaced G runs in the sense strand (PQS3+, upper row) and antisense strand (PQS3-, lower row) as identified by pqsfinder where (a) score > 64 and (b) score > 25. Gypsy (RLG), Copia (RLC) and other (RLX) superfamilies are shown in separate columns. Frequency (vertical axis) represents the number of PQS present in a window covering 2% of TE length. 75% of LTRs fall within the black rectangles shown below the horizontal axis (3rd quartile = 0.125; mean LTR length = 0.100; maximum length = 0.427). (TIFF 3702 kb) [file 12864_2018_4563_MOESM3_ESM.tif]

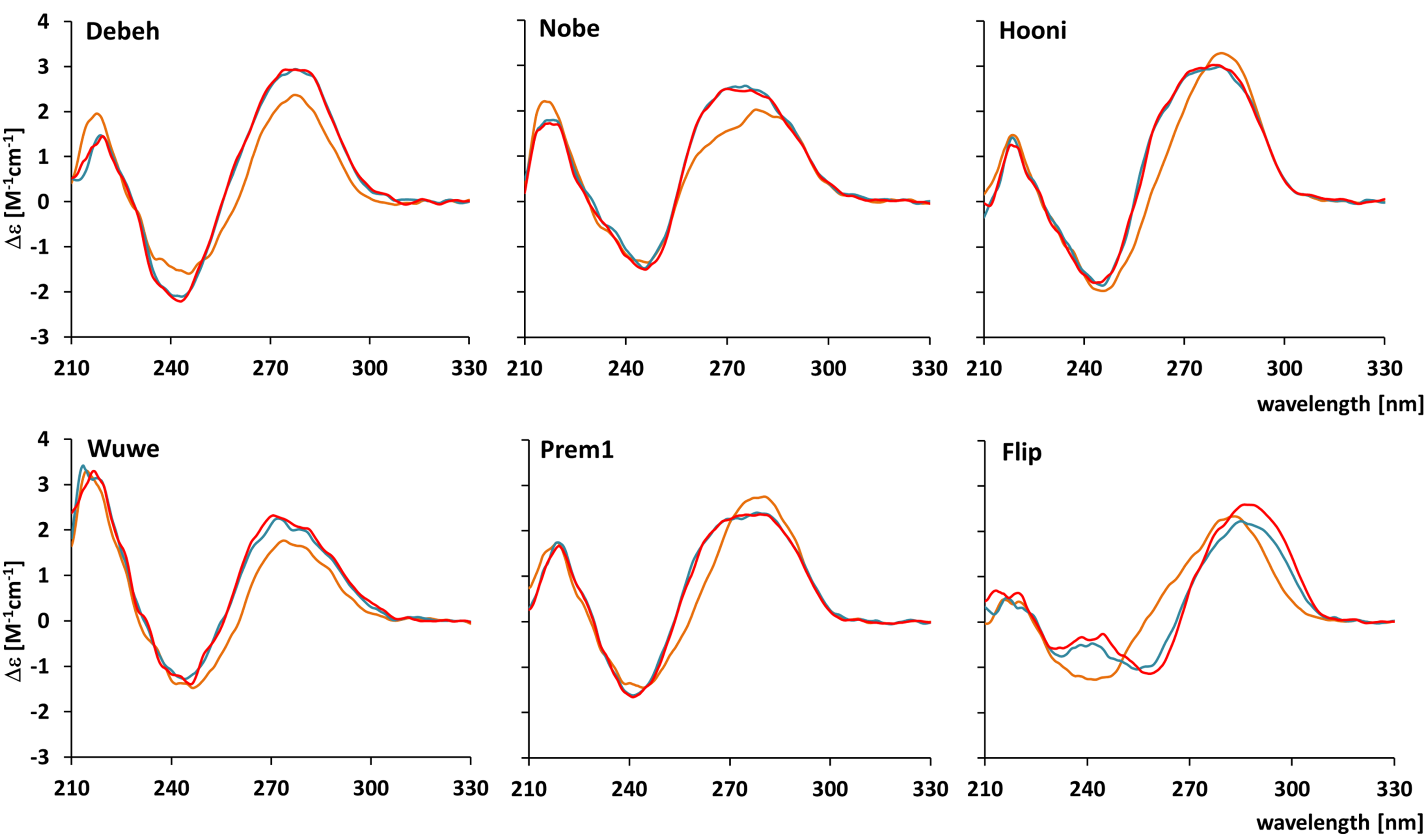

Supplement: Supplementary file 5 — CD spectra of oligonucleotides without G4-forming ability. CD spectra of oligonucleotides representing wild-type PQS from various LTR retrotransposons obtained at different concentrations of potassium ions (orange: 0 mM K+; blue: 150 mM K+ and red: 150 mM K+ after annealing). Debeh, Nobe, Hooni, Wuwe and Prem1 are oligonucleotides with long middle loop. Flip has short middle loop. (TIFF 883 kb) [file 12864_2018_4563_MOESM5_ESM.tif]
